# Impaired Carbohydrate Digestion and Transport and Mucosal Dysbiosis in the Intestines of Children with Autism and Gastrointestinal Disturbances

**DOI:** 10.1371/journal.pone.0024585

**Published:** 2011-09-16

**Authors:** Brent L. Williams, Mady Hornig, Timothy Buie, Margaret L. Bauman, Myunghee Cho Paik, Ivan Wick, Ashlee Bennett, Omar Jabado, David L. Hirschberg, W. Ian Lipkin

**Affiliations:** 1 Center for Infection and Immunity, Columbia University, New York, New York, United States of America; 2 Division of Pediatric Gastroenterology and Nutrition, Massachusetts General Hospital, Boston, Massachusetts, United States of America; 3 Department of Neurology, Harvard Medical School and Departments of Neurology and Pediatrics and Learning and Developmental Disabilities Evaluation and Rehabilitation Services (LADDERS), Massachusetts General Hospital, Boston, Massachusetts, United States of America; 4 Department of Biostatistics, Columbia University, Mailman School of Public Health, New York, New York, United States of America; National Institutes of Health, United States of America

## Abstract

Gastrointestinal disturbances are commonly reported in children with autism, complicate clinical management, and may contribute to behavioral impairment. Reports of deficiencies in disaccharidase enzymatic activity and of beneficial responses to probiotic and dietary therapies led us to survey gene expression and the mucoepithelial microbiota in intestinal biopsies from children with autism and gastrointestinal disease and children with gastrointestinal disease alone. Ileal transcripts encoding disaccharidases and hexose transporters were deficient in children with autism, indicating impairment of the primary pathway for carbohydrate digestion and transport in enterocytes. Deficient expression of these enzymes and transporters was associated with expression of the intestinal transcription factor, CDX2. Metagenomic analysis of intestinal bacteria revealed compositional dysbiosis manifest as decreases in Bacteroidetes, increases in the ratio of Firmicutes to Bacteroidetes, and increases in Betaproteobacteria. Expression levels of disaccharidases and transporters were associated with the abundance of affected bacterial phylotypes. These results indicate a relationship between human intestinal gene expression and bacterial community structure and may provide insights into the pathophysiology of gastrointestinal disturbances in children with autism.

## Introduction

Autism spectrum disorders (ASD) are defined by impairments in verbal and non-verbal communication, social interactions, and repetitive and stereotyped behaviors. In addition to these core deficits, previous reports indicate that the prevalence of gastrointestinal (GI) symptoms ranges widely in individuals with ASD, from 9 to 91% in different study populations [1]. Macroscopic and histological observations in ASD include findings of ileo-colonic lymphoid nodular hyperplasia, enterocolitis, gastritis, and esophagitis [2], [3], [4], [5], [6], [7]. Associated changes in intestinal inflammatory parameters include higher densities of lymphocyte populations, aberrant cytokine profiles, and deposition of immunoglobulin (IgG) and complement C1q on the basolateral enterocyte membrane [5], [8], [9], [10], [11], [12]. Reported functional disturbances include increased intestinal permeability [13], deficient enzymatic activity of disaccharidases [7], increased secretin-induced pancreatico-biliary secretion [7], and abnormal fecal Clostridia taxa [14], [15], [16]. Some children placed on exclusion diets or treated with the antibiotic vancomycin are reported to improve in cognitive and social function [17], [18]. Furthermore, a recent study found a strong correlation between GI symptoms and autism severity [19].

The intestinal mucoepithelial layer must maximize nutritional uptake of dietary components while maintaining a barrier to toxins and infectious agents. Although some aspects of these functions are host-encoded, others are acquired through symbiotic relationships with microbial flora. Dietary carbohydrates enter the intestine as monosaccharides (glucose, fructose, and galactose), disaccharides (lactose, sucrose, and maltose), or complex polysaccharides. Following digestion with salivary and pancreatic amylases, carbohydrates are further digested by disaccharidases expressed by absorptive enterocytes in the brush border of the small intestine and transported as monosaccharides across the intestinal epithelium. Although humans lack the glycoside hydrolases and polysaccharide lyases necessary for cleavage of glycosidic linkages present in plant cell wall polysaccharides, oligosaccharides, storage polysaccharides, and resistant starches, intestinal bacteria encoding these enzymes expand our capacity to extract energy from dietary polysaccharides [20], [21]. As an end product of polysaccharide fermentation, bacteria produce short-chain fatty acids (butyrate, acetate, and propionate) that serve as energy substrates for colonocytes, modulate colonic pH, regulate colonic cell proliferation and differentiation, and contribute to hepatic gluconeogenesis and cholesterol synthesis [22], [23]. Intestinal microbes also mediate postnatal development of the gut mucoepithelial layer, provide resistance to potential pathogens, regulate development of intraepithelial lymphocytes and Peyer's patches, influence cytokine production and serum immunoglobulin levels, promote systemic lymphoid organogenesis, and influence brain development and behavior [24], [25], [26].

Although bacteria have been examined in fecal material from children with autism, no study to date has reported analyses of microbiota adherent to the intestinal mucoepithelium. Furthermore, there are no reports wherein intestinal gene expression in children with autism has been correlated with alterations in intestinal microbiota. GI dysfunction is commonly reported in children with autism; however, it remains unclear how or whether GI dysfunction in children with autism differs from GI dysfunction found in typically developing children. Here we investigate expression of human genes involved in carbohydrate digestion and transport along with bacterial community composition in intestinal biopsies from children with autistic disorder and GI disease (AUT-GI) compared to children with GI disease alone (Control-GI). Results from gene expression assays and metagenomic analysis of over half a million bacterial 16S rRNA gene sequences revealed decreased mRNA expression for human disaccharidases and hexose transporters and compositional dysbiosis in children in the AUT-GI group compared to those in the Control-GI group. These results highlight the complex relationship between human intestinal gene expression and bacterial community structure and provide insights into the molecular mechanisms underlying the pathophysiology of gastrointestinal disturbances in children with autism.

## Results

### Patient Characteristics

All AUT-GI and Control-GI children evaluated were male (**Table 1**). Mean onset age for autism in AUT-GI was 13.4+/−5.4 months. Median age at biopsy was similar for AUT-GI and Control-GI children [median age in years (interquartile range, IQR), AUT-GI, 4.5 (1.3); and Control-GI, 4.0 (1.1)]. Median number of medications used and the IQR for number of medications used per subject were identical in AUT-GI and Control-GI children. Food allergies (FA) were commonly reported in both AUT-GI (67%) and Control-GI (71%) subjects. The majority of children with FA had reported milk-related allergy (90% for AUT-GI and 100% for Control-GI) and/or wheat-related allergy (80% for AUT-GI and 80% for Control-GI). Beneficial effects of dietary intervention on GI disturbances were reported for all AUT-GI and Control-GI subjects with FA. Comorbid conditions were reported in 67% of AUT-GI children and 100% of Control-GI children. The most commonly reported comorbid conditions were atopic manifestations (asthma, atopic dermatitis, and allergic rhinitis). Atopic manifestations were more common in Control-GI children (100%) than AUT-GI children (53%) (**Table 1**). The frequency of individual atopic manifestations was higher in Control-GI children. The largest difference in frequency was for asthma, which was only reported in 20% of AUT-GI children compared to 71% of Control-GI children (**Table 1**). Established intestinal disorders were only reported in a few subjects: two AUT-GI subjects (13%: 1 with IBD, 1 with Celiac disease) and one Control-GI subject (14%: IBD). For detailed information related to medication use, food allergy, and comorbid conditions in individual AUT-GI and Control-GI children see **Table S1**. The prevalence of specific GI symptoms was similar in AUT-GI and Control-GI children (**Table 2**). The most frequently reported GI symptoms in both groups were diarrhea (AUT-GI, 80%; Control-GI, 71%) and changes in stool frequency (AUT-GI, 87%; Control-GI, 71%) and consistency (AUT-GI, 80%; Control-GI, 86%). Mucus in stool was more frequent in Control-GI (86%) compared to AUT-GI (40%) children; bloating was more frequent in AUT-GI (60%) compared to Control-GI (29%) children. Regression (loss of language and/or other skills following acquisition) is reported in 20% to 40% of individuals with autism, and some studies suggest higher rates of GI symptoms in ASD subjects with regression than those without regression [27]. Eighty-seven percent of AUT-GI subjects in our study had behavioral regression (**Table S2**).

**Table 1:** Summary of patient characteristics.

Subject Characteristic	Subcategory	AUT-GI (n = 15)	Control-GI (n = 7)
**Autism onset age in months, mean ± SD**	AUT-GI subjects	13.4±5.4	-
**Gender**	All subjects	All male	All male
**Ethnicity, n (%)**	Caucasian	14 (93)	6 (86)
	Hispanic	1 (7)	0 (0)
	African-American	0 (0)	1 (14)
**Age at biopsy in years, median (IQR) [range]**	All subjects	4.5 (1.3) [3.5–5.9]	4.0 (1.1) [3.9–5.5]
**Medications-number per subject** a **, median (IQR) [range]**	All subjects	5 (7) [1]–[21]	5 (7) [0–8]
**Food allergies, n (% of subjects)**	All subjects	10 (67)	5 (71)
**Milk-related allergy** b **, n (% of subjects with food allergy)**	Subjects reporting any food allergy	9 (90)	5 (100)
**Wheat-related allergy** c **, n (% of subjects with food allergy)**	Subjects reporting any food allergy	8 (80)	4 (80)
**Diet improvement of GI problems, n (% of subjects with food allergy)**	Subjects reporting any food allergy	10 (100)	5 (100)
**Current comorbid conditions-number per subject, median (IQR) [range]**	All subjects	1 (1.75) [0–5]	2 (2.75) [1]–[6]
**Comorbid atopic disease manifestations** d **, n (% of subjects)**	All subjects	8 (53)	7 (100)
**Asthma, n (% of subjects)**	All subjects	3 (20)	5 (71)
**Atopic dermatitis, n (% of subjects)**	All subjects	4 (27)	4 (57)
**Allergic rhinitis, n (% of subjects)**	All subjects	4 (27)	3 (43)

a – Number of prescription drugs and alternative agents taken regularly, per subject.

b – Allergy to milk, casein, lactose, or dairy.

c – Allergy to wheat or gluten.

d – Asthma, Allergic rhinitis, or Atopic dermatitis.

**Table 2:** Summary of patient GI symptoms.

GI Symptoms	AUT-GI, n (%)	Control-GI, n (%)
**Diarrhea**	12 (80)	5 (71)
**Diarrhea w/Vomiting**	2 (13)	2 (29)
**Vomiting**	2 (13)	1 (14)
**Bloating**	9 (60)	2 (29)
**Δ Stool Frequency**	13 (87)	5 (71)
**Δ Stool Consistency**	12 (80)	6 (86)
**Mucus in Stool**	6 (40)	6 (86)
**Blood in Stool**	2 (13)	1 (14)
**Pain**	8 (53)	5 (71)
**Weight Loss**	3 (20)	0 (0)
**Fever**	1 (7)	0 (0)

### Deficient ileal mRNA expression of disaccharidases and hexose transporters in AUT-GI children

**Figure 1:**
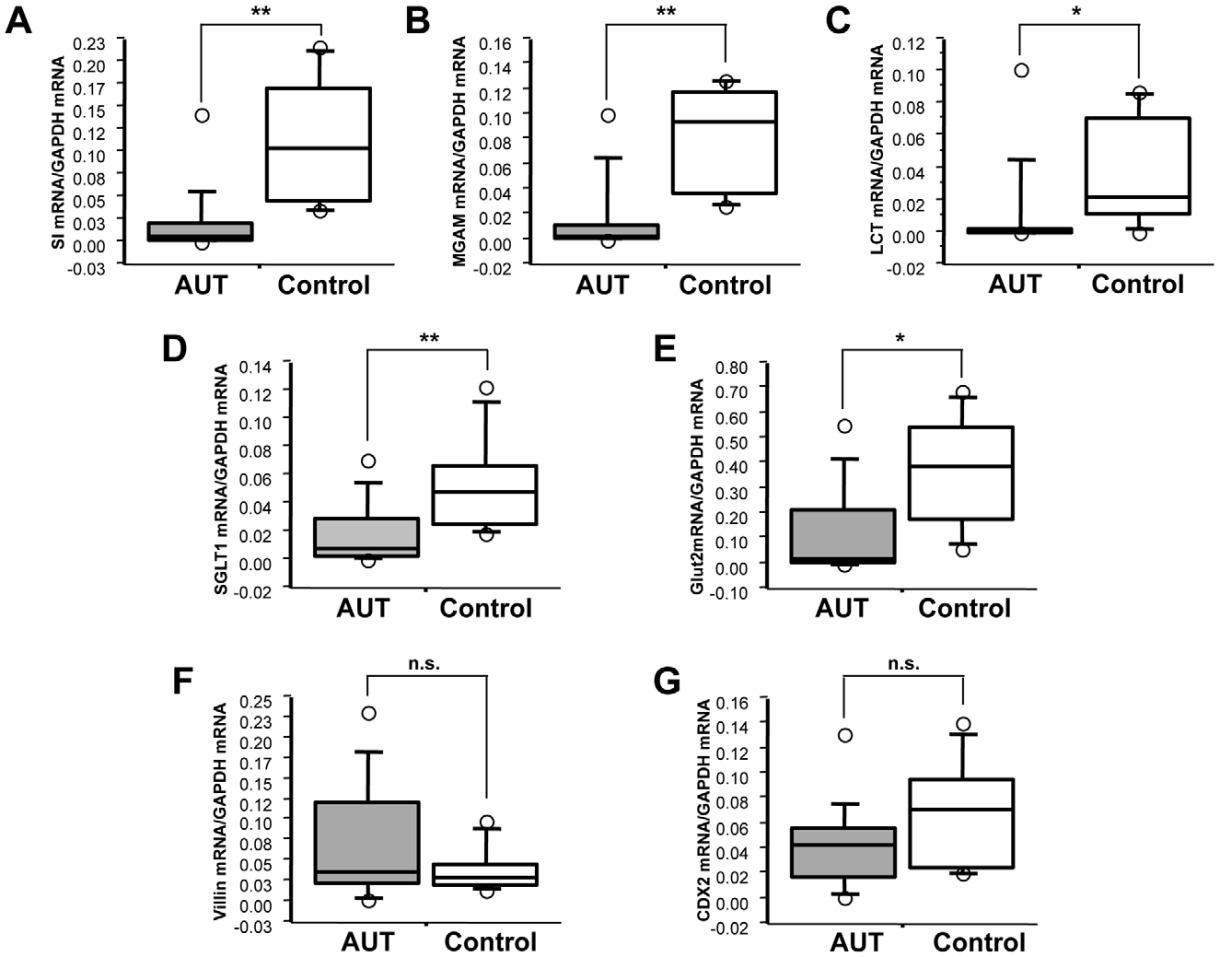
Quantitative real-time PCR analysis of disaccharidases, hexose transporters, villin and CDX2 transcripts. Box-and-whisker plots displaying (**A**) SI (Mann-Whitney; *p* = 0.001), (**B**) MGAM (Mann-Whitney; *p* = 0.003), (**C**) LCT (Mann-Whitney; *p* = 0.032), (**D**) SGLT1 (Mann-Whitney; *p* = 0.008), (**E**) GLUT2 (Mann-Whitney; *p* = 0.010), (**F**) Villin (Mann-Whitney; *p* = 0.307), and (**G**) CDX2 (Mann-Whitney; *p* = 0.192) mRNA expression normalized to GAPDH mRNA in ileal biopsies from AUT-GI (AUT) and Control-GI (Control) patients. *, *p*<0.05; **, *p*<0.01; n.s., not significant.

**Figure 2:**
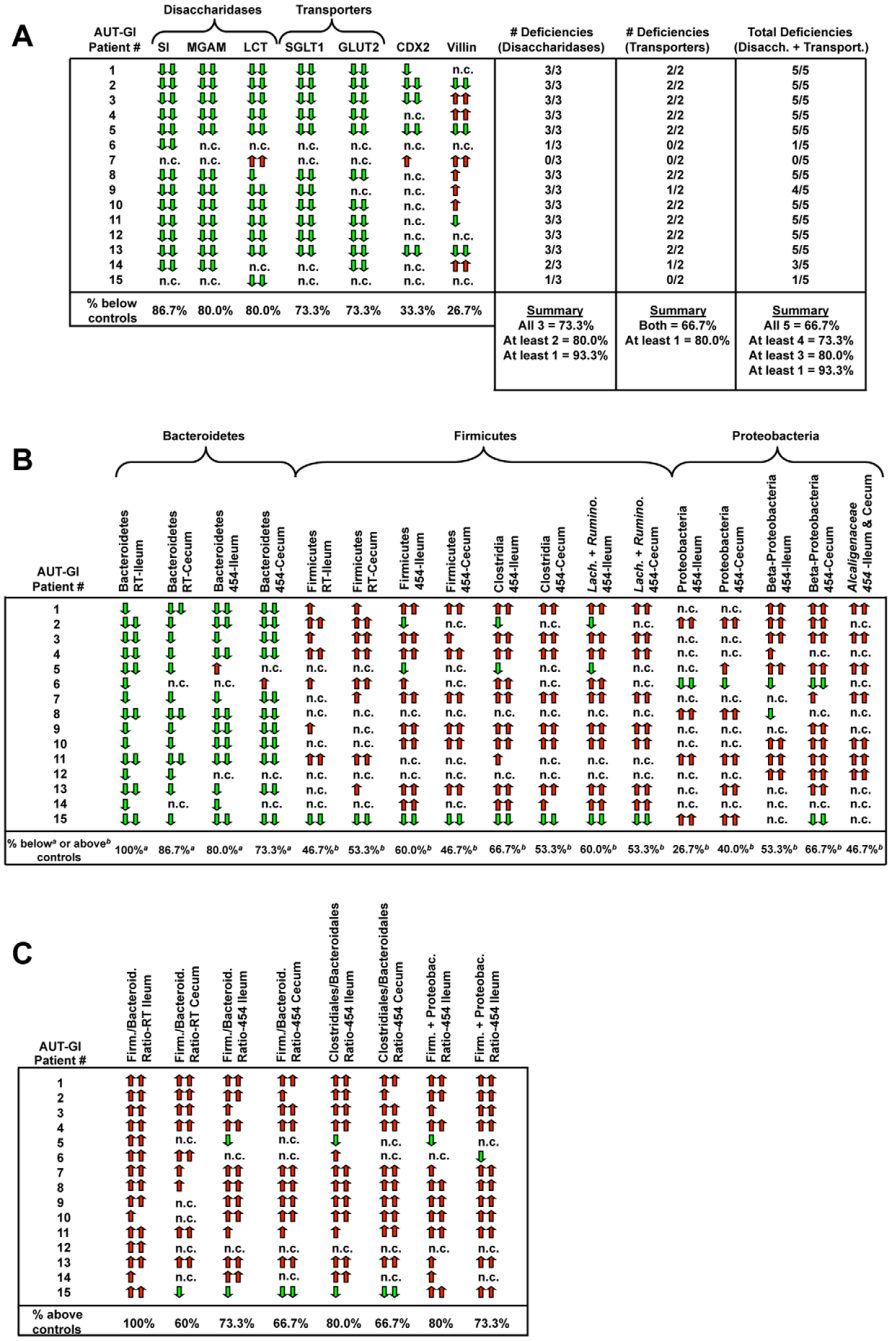
Patient summary tables for gene expression and bacterial assays. (**A–C**) Increases or decreases in AUT-GI children in both gene expression (**A**) and bacterial parameters (**B** and **C**) were determined for each individual based on the levels of each parameter in the Control-GI group. (**A**) The gene expression levels in the AUT-GI children that exceeded the 75^th^ percentile of Control-GI values and were at least 2-fold increased relative to the Control-GI mean (red arrow) or below the 25^th^ percentile of Control-GI values and at least 2-fold decreased relative to the Control-GI mean (green arrow) were scored as an increase or decrease, respectively. Values above the 90^th^ or below the 10^th^ percentiles of Control-GI children are indicated by double arrows. (**B** and **C**) Bacterial parameters in AUT-GI children that exceeded the 75^th^ percentile of Control-GI values (red arrows) or were below the 25^th^ percentile of Control-GI values (green arrows) were scored as an increase or decrease, respectively. Values above the 90^th^ or below the 10^th^ percentiles of Control-GI children are indicated by double arrows. Results are shown for data obtained by real-time PCR (RT), where performed, and pyrosequencing (454). (n.c. = no change relative to defined cut-off values for Control-GI children).

We examined transcript levels for three primary brush border disaccharidases (sucrase isomaltase [SI], maltase glucoamylase [MGAM], and lactase [LCT]) in ileal biopsies of AUT-GI and Control-GI children by real-time PCR. Levels of mRNA for all three enzymes were significantly decreased in AUT-GI children: SI (**Figure 1A**: Mann-Whitney, p = 0.001), MGAM (**Figure 1B**: Mann-Whitney, p = 0.003) and LCT (**Figure 1C**: Mann-Whitney, p = 0.032). Within the AUT-GI group, 86.7%, 80%, and 80% of children had deficient transcript levels (defined as below the 25^th^ percentile of values obtained for Control-GI children and at least two-fold below Control-GI mean values) for SI, MGAM, and LCT, respectively (**Figure 2A** and **Table S3**). Nearly all (14/15, or 93.3%) AUT-GI children had deficiencies in at least one disaccharidase enzyme; 80% had deficiencies in 2 or more enzymes; 73.3% had deficiencies in all three enzymes (**Figure 2A**). Deficiencies in LCT mRNA in AUT-GI children were not attributable to disproportionate adult-type hypolactasia genotypes in the AUT-GI group relative to the Control-GI group (**Figure S1A–D** and **Text S1**).

Two hexose transporters, sodium-dependent glucose cotransporter (SGLT1) and glucose transporter 2 (GLUT2), mediate transport of monosaccharides in the intestine. SGLT1, located on the luminal membrane of enterocytes, is responsible for the active transport of glucose and galactose from the intestinal lumen into enterocytes. GLUT2 transports glucose, galactose, and fructose across the basolateral membrane into the circulation and may also translocate to the apical membrane [28]. Real-time PCR revealed a significant decrease in ileal SGLT1 mRNA (**Figure 1D**: Mann-Whitney, p = 0.008) and GLUT2 mRNA (**Figure 1E**: Mann-Whitney, p = 0.010) in AUT-GI children. For SGLT1, 73.3% of AUT-GI children had deficient transcript levels, and 73.3% of AUT-GI children had deficient GLUT2 transcript levels, relative to Control-GI children (**Figure 2A**). Deficiencies were found in at least one hexose transporter in 80% of AUT-GI children; 66.7% had deficiencies in both transporters.

In total, 93.3% (14/15) of AUT-GI children had mRNA deficiencies in at least one of the 5 genes involved in carbohydrate digestion or transport; 66.7% (10/15) had mRNA deficiencies in all 5 genes (**Figure 2A**).

To determine whether reductions in disaccharidase and transporter transcript levels reflected loss of or damage to intestinal epithelial cells, we measured mRNA levels associated with a tissue-specific marker restricted to these cells, villin [29], [30]. Ileal villin mRNA levels were not decreased in AUT-GI children (Mann-Whitney, p = 0.307) (**Figure 1F**). Normalization of SI, MGAM, LCT, SGLT1, and GLUT2 to villin mRNA levels did not correct deficits (**Figure S2A–E**).

The transcription factor, caudal type homeobox 2 (CDX2), regulates expression of SI, LCT, GLUT2, and SGLT1 [31], [32], [33], [34]. Real-time PCR experiments demonstrated lower levels of CDX2 mRNA in some AUT-GI subjects versus controls; however, group differences were not significant (**Figure 1G**: Mann-Whitney, p = 0.192). Although only 33.3% of AUT-GI patients had deficient CDX2 mRNA levels (**Figure 2A**), 86.7% of AUT-GI children had CDX2 levels below the 50^th^ percentile of Control-GI children, and 46.7% of AUT-GI children had at least a two-fold decrease in CDX2 expression relative to the Control-GI mean. Only one AUT-GI child (patient #7) had CDX2 levels above the 75^th^ percentile of Control-GI children and a near two-fold (1.95-fold) increase in CDX2 expression (**Figure 2A** and **Table S3**). This child was the only AUT-GI subject who did not show signs of deficiencies in disaccharidases or transporters.

AUT-GI children with deficiencies in all five disaccharidases and transporters had significantly lower levels of CDX2 mRNA compared to AUT-GI children with fewer than five deficiencies (**Figure S2F**: Mann-Whitney, p = 0.037). However, only a trend toward decreased CDX2 levels was found when comparing AUT-GI children with deficiencies in all five disaccharidases and transporters and Control-GI children (**Figure S2F**: Mann-Whitney, p = 0.064).

Multiple linear regression analyses were conducted to determine whether diagnostic status (AUT-GI or Control-GI), CDX2 mRNA expression, or villin mRNA expression (predictor variables) was associated with mRNA expression levels of individual disaccharidases (SI, MGAM, and LCT) or transporters (SGLT1 and GLUT2) (**Table 3**). In each of the five models, where the expression of SI, MGAM, LCT, SGLT1, or GLUT2 served as outcome variables, CDX2 contributed significantly to the model. As the level of CDX2 increased by one unit of standard deviation, there was a concomitant approximate one unit increase in log-transformed disaccharidase and transporter transcript levels (ranging from 0.78 for SGLT1 to 1.30 for LCT). None of the interaction terms between CDX2 and diagnostic status were significant, implying that the magnitude of the effect of CDX2 on log-transformed enzyme and transporter levels was the same for AUT-GI and Control-GI children. For SGLT1 and GLUT2 expression, CDX2 was the sole significant predictor variable in the model. Diagnostic status and CDX2 were significant predictors of SI, MGAM, and LCT expression, suggesting that additional factors associated with diagnostic status must also contribute to expression levels for these enzymes. Villin was not a significant predictor of the expression levels of any of the five genes after adjusting for CDX2.

**Table 3:** Multiple linear regression analysis examining CDX2 and villin as predictors of disaccharidase and transporter mRNA expression among AUT-GI and Control-GI children.

			Predictor Variables: Coefficient Estimate		
Outcome Variable	F_3,18_ (p-value)	Adjusted R^2^	Diagnostic Status	CDX2STDev	VillinSTDev
**SI**	10.35 (0.0003)***	0.57	−1.83*	0.93*	−0.19
**MGAM**	8.78 (0.0008)***	0.53	−2.10*	1.15*	−0.20
**LCT**	10.87 (0.0003)***	0.59	−2.25*	1.30*	0.65
**SGLT1**	6.88 (0.0030)**	0.46	−1.36†	0.78*	0.12
**GLUT2**	6.06 (0.0050)**	0.42	−1.90†	1.06*	0.03

STDev – Change in log-transformed outcome variable levels per unit standard deviation increase in predictor variable.

*, p<0.05;

**, p<0.01;

***, p<0.001;

†, p<0.1 (trend).

### Mucosal dysbiosis in AUT-GI children

To determine whether deficient carbohydrate digestion and absorption influenced the composition of intestinal microflora, ileal and cecal biopsies from AUT-GI and Control-GI children were analyzed by bacterial 16S rRNA gene pyrosquencing. The use of biopsies rather than fecal material allowed us to assess the mucoepithelia-associated microbiota, as these likely establish more intimate interactions with the human intestinal epithelium and immune cells [35]. A total of 525,519 bacterial sequences were subjected to OTU (Operational Taxonomic Unit; defined at 97% identity) analysis and classified with RDP (Ribosomal Database Project). Rarefaction analysis of OTUs did not suggest a loss or gain of overall diversity based on Shannon Diversity estimates in AUT-GI compared to Control-GI children (See **Figure S3A–D** and **Text S1**).

**Figure 3:**
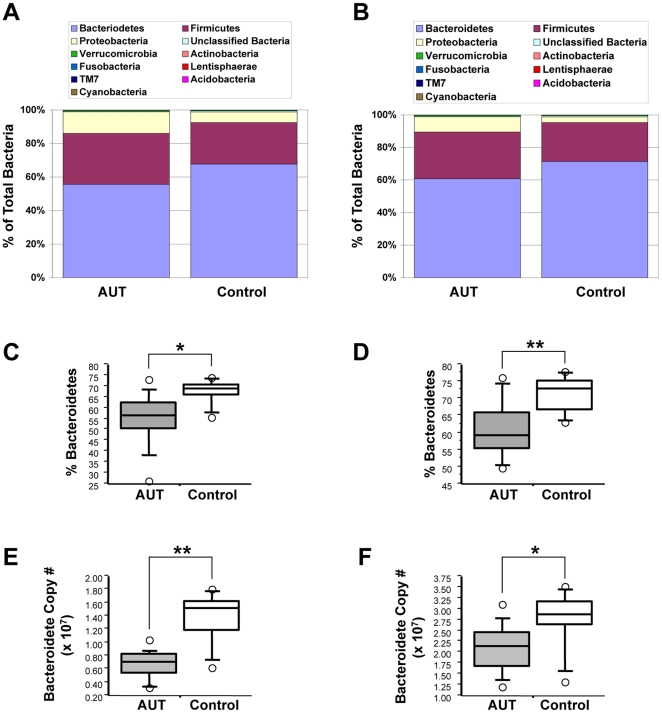
Composition of intestinal microflora in AUT-GI children. (**A–B**) Phylum-level comparison of the average relative abundance of bacterial taxa in ileal (**A**) and cecal (**B**) biopsies. (**C–D**) Bacteroidete abundance, obtained from pyrosequencing for ileal (**C**; Mann-Whitney, *p* = 0.012) and cecal (**D**; Mann-Whitney, *p* = 0.008) biopsies. (**E–F**) Bacteroidete-specific quantitative real-time PCR analysis of ileal (**E**; Mann-Whitney, *p* = 0.003) and cecal (**F**; Mann-Whitney, *p* = 0.022) biopsies; copy number values are normalized relative to total bacteria copy numbers. *, *p*<0.05; **, *p*<0.01.

Classification of pyrosequencing reads revealed that Bacteroidetes and Firmicutes were the most prevalent taxa in ileal and cecal tissues of AUT-GI and Control-GI children, followed by Proteobacteria (**Figure 3A, B**). Other phyla identified at lower levels included Verrucomicrobia, Actinobacteria, Fusobacteria, Lentisphaerae, and TM7, as well as “unclassified bacteria” (sequences that could not be assigned at the phylum-level) (**Figure 3A, B**). The abundance of Bacteroidetes was lower in AUT-GI ileal (**Figure 3C**: Mann-Whitney, p = 0.012) and cecal biopsies (**Figure 3D**: Mann-Whitney, p = 0.008), as compared with the abundance of Bacteroidetes in Control-GI biopsies. Real-time PCR using Bacteroidete-specific primers confirmed decreases in Bacteroidetes in AUT-GI ilea (**Figure 3E**: Mann-Whitney, p = 0.003; **Table S4:** 50% average reduction in Bacteroidete 16S rDNA copies; range, 24.36% to 76.28% decrease) and ceca (**Figure 3F**: Mann-Whitney, p = 0.022; **Table S4:** 29% average reduction in 13 of 15 patients with reduced Bacteroidetes; range, 7.22% to 56.54% decrease), with levels below the 25^th^ percentile of Control-GI children in 100% of AUT-GI ilea and 86.7% of AUT-GI ceca (**Figure 2B**). OTU analysis of Bacteroidete sequences suggested that deficiencies in Bacteroidete sequences in AUT-GI subjects were attributable to cumulative losses of 12 predominant phylotypes of Bacteroidetes, rather than loss of any one specific phylotype (**Figure S4A–E** and **Text S1**).

**Figure 4:**
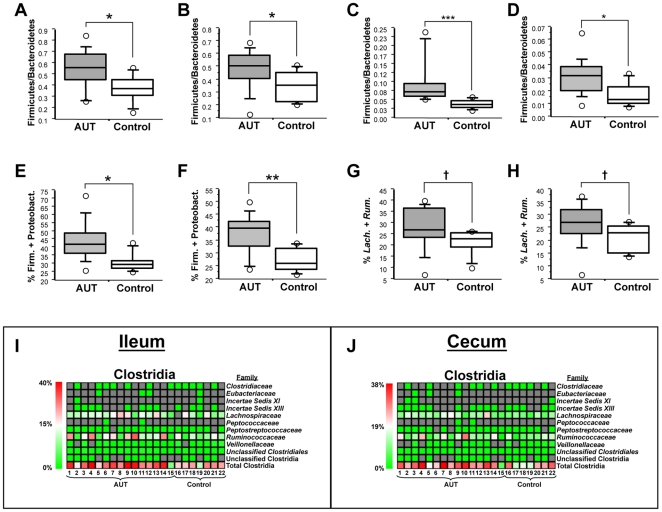
Firmicute/Bacteroidete ratios, Clostridia family abundance, and cumulative levels of Firmicutes and Proteobacteria. (**A–B**) Firmicute/Bacteroidete ratio from pyrosequencing reads obtained from ileal (**A**; Mann-Whitney, *p* = 0.026) and cecal (**B**; Mann-Whitney, *p* = 0.032) biopsies. (**C–D**) Firmicute/Bacteroidete ratios obtained by real-time PCR for ilea (**C**; Mann-Whitney, p = 0.0006) and ceca (**D**; Mann-Whitney, p = 0.022). (**E–F**) Cumulative abundance of Firmicutes and Proteobacteria from ileal (**E**; Mann-Whitney, *p* = 0.015) and cecal (**F**; Mann-Whitney, *p* = 0.007) biopsies. (**G–H**) Cumulative levels of members of the families *Lachnospiraceae* and *Ruminococcaceae* in ileal (**G**; Mann-Whitney; *p* = 0.062) and cecal (**H**; Mann-Whitney; *p* = 0.098) biopsies. (**I–J**) Family-level abundance distributions of the class Clostridia in ileum (**I**) and cecum (**J**): bottom row displays cumulative levels of all family members by patient; gray cells indicate where no sequences were observed for a given taxa. *, *p*<0.05; **, *p*<0.01; ***, p<0.001; †, *p*<0.1 (trend).

Analysis of pyrosequencing reads revealed a significant increase in Firmicute/Bacteroidete ratios in AUT-GI ilea (**Figure 4A**: Mann-Whitney, p = 0.026) and ceca (**Figure 4B**: Mann-Whitney, p = 0.032). An increase was also observed at the order level for Clostridiales/Bacteroidales ratios in ilea (**Figure S5A**: Mann-Whitney, p = 0.012) and ceca (**Figure S5B**: Mann-Whitney, p = 0.032). Real-time PCR using Firmicute- and Bacteroidete-specific primers confirmed increases in Firmicute/Bacteroidete ratios in AUT-GI ilea (**Figure 4C**: Mann-Whitney, p = 0.0006) and ceca (**Figure 4D**: Mann-Whitney, p = 0.022). Based on real-time PCR results, Firmicute/Bacteroidete ratios were above the 75^th^ percentile of Control-GI values in 100% of AUT-GI ilea and 60% of AUT-GI ceca (**Figure 2C**).

**Figure 5:**
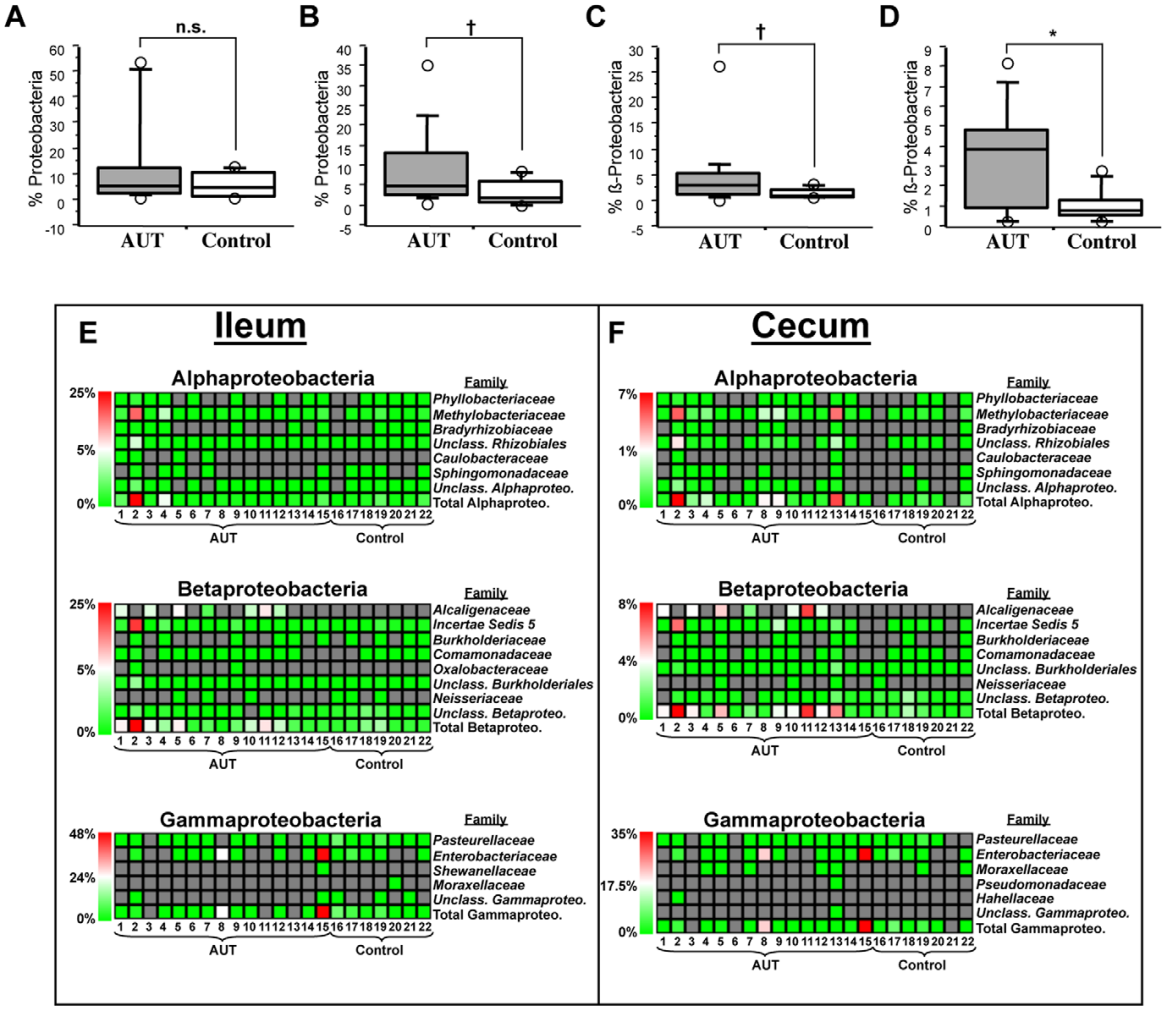
Abundance of Proteobacteria in AUT-GI and Control-GI children. (**A–B)** Phyla-level abundance of Proteobacteria members in ileal (**A**; Mann-Whitney, *p* = 0.549) and cecal (**B**; Mann-Whitney, *p* = 0.072) biopsies obtained by pyrosequencing. (**C–D**) Class-level abundance of Betaproteobacteria members in ileal (**C**; Mann-Whitney, *p* = 0.072) and cecal (**D**; Mann-Whitney, *p* = 0.038) biopsies. (**E–F**) Family-level abundance distributions of bacteria within the classes Alpha-, Beta-, and Gammaproteobacteria in ileal (**E**) and cecal (**F**) biopsies: bottom row of each heatmap displays the cumulative levels of family members in each class of Proteobacteria by patient; gray cells indicate where no sequences were identified for a given taxa. *, *p*<0.05; †, *p*<0.1 (trend); n.s., not significant.

The cumulative level of Firmicutes and Proteobacteria was significantly higher in the AUT-GI group in both ileal (**Figure 4E**: Mann-Whitney, p = 0.015) and cecal (**Figure 4F**: Mann-Whitney, p = 0.007) biopsies; however, neither Firmicute nor Proteobacteria levels showed significant differences on their own (**Figure S5C–F** and **Figure 5A, B**). These results suggest that the observed decrease in Bacteroidetes in AUT-GI children is accompanied by an increase in Firmicutes (Ileal biopsies- patients #1, 3, 4, 6, 7, 9, 10, 13, and 14; Cecal biopsies- patients #1, 3, 4, 7, 9, 10, and 13), or Proteobacteria (Ileal biopsies- patients #2, 8, 11 and 15; Cecal biopsies- patients #2, 5, 8, 11, 13, and 15), or both (Cecal biopsies- patient #13) (**Figure 2B** and **Figure S6A, B**).

Within the Firmicute phyla, order-level analysis of pyrosequencing reads indicated trends toward increases in Clostridiales in AUT-GI ilea (**Figure S5G**: Mann-Whitney, p = 0.072) and ceca (**Figure S5H**: Mann-Whitney, p = 0.098). Family-level analysis revealed that increased Clostridiales levels in AUT-GI patient samples were largely attributable to increases in *Lachnospiraceae* and *Ruminococcaceae* (**Figure 4G–J**). Cumulative levels of *Lachnospiraceae* and *Ruminococcaceae* above the 75^th^ percentile of the corresponding levels in Control-GI samples were found in 60% of AUT-GI ileal and 53.3% of AUT-GI cecal samples (**Figure 2B**). Genus-level analysis indicated that members of the genus *Faecalibacterium* within the family *Ruminococcaceae* contributed to the overall trend toward increased Clostridia levels (**Figure S7A, B**). Within *Lachnospiraceae*, members of the genus *Lachnopsiraceae Incertae Sedis*, *Unclassified Lachnospiraceae*, and, to a lesser extent, *Bryantella* (cecum only), contributed to the overall trend toward increased Clostridia (**Figure S7A, B**).

Within the Proteobacteria phyla, class-level abundance of Betaproteobacteria tended to be higher in the ilea of AUT-GI patients (**Figure 5C**: Mann-Whitney, p = 0.072); significantly higher abundance of Betaproteobacteria were found in AUT-GI ceca (**Figure 5D**: Mann-Whitney, p = 0.038). Levels of Betaproteobacteria were above the 75^th^ percentile of Control-GI children in 53.3% of AUT-GI ilea and 66.7% of AUT-GI ceca (**Figure 2B**). Family-level analysis revealed that members of the families *Alcaligenaceae* (patients #1, 3, 5, 7, 10, 11, and 12) and *Incertae Sedis 5* (patient #2 only) contributed to the increases in Betaproteobacteria in ilea (**Figure 5E**) and ceca (**Figure 5F**). *Alcaligenaceae* sequences were detected in 46.7% of AUT-GI children and none of the Control-GI children. Elevated levels of Proteobacteria in AUT-GI ilea and ceca reflected increased Alpha- (families *Methylobacteriaceae* and *Unclassified Rhizobiales*) and Betaproteobacteria (family *Incertae Sedis 5*) for patient #2 and increased Gammaproteobacteria (family *Enterobacteriaceae*) for patients #8 and #15 (**Figure 5E, F**). Levels of Alpha-, Delta-, Gamma-, and Epsilonproteobacteria were not significantly different between AUT-GI and Control-GI samples (data not shown).

The use of probiotics, proton-pump inhibitors, or antibiotics has been shown to impact the intestinal microbiome [36], [37], [38]. Analysis of the potential effects of these agents in this cohort revealed only one potential confounding effect: a correlation between the ratio of Firmicutes to Bacteroidetes in the cecum obtained by real-time PCR in AUT-GI children who had taken probiotics (**Table S5A** and **Text S1**). No effect of proton-pump inhibitors was observed for any of the significant variables assessed in this study (**Table S5B** and **Text S1**). Only one patient, a control (Control-GI patient #16), had taken an antibiotic (amoxicillin) in the three months prior to biopsy (See **Table S5C** and **Text S1**).

### Disaccharidase and transporter mRNA levels as predictors of bacterial abundance

Multiple linear regression analyses were conducted to determine whether diagnostic status (AUT-GI or Control-GI) and mRNA expression of disaccharidases (SI, MGAM, and LCT) and transporters (SGLT1 and GLUT2) (predictor variables) were associated with bacterial levels as outcome variables (**Table 4**). For Bacteroidetes, SGLT1 (ileum and cecum) and SI (cecum only) were significant predictors. In both the ileum and cecum, Bacteroidete levels increased as SGLT1 transcript levels increased. In the cecum, Bacteroidete levels significantly decreased as the levels of SI increased (a similar marginal effect was observed in ileum). Bacteroidete levels were lower among AUT-GI children compared to Control-GI children even after adjusting for the expression of all disaccharidases and transporters.

**Table 4:** Multiple linear regression analysis examining disaccharidases and transporters as predictors of bacterial levels among AUT-GI and Control-GI children.

			Main Effects: Coefficient Estimate						
Outcome Variable	F-statistic (p-value)	Adjusted R^2^	Diagnostic Status	SISTDev	MGAMSTDev	LCTSTDev	SGLT1STDev	GLUT2STDev	Interaction Terms with Status (CoefficientSTDev)
**Bacteroidetes, Ileum-RT**	5.52^a^ (0.003)**	0.56	−0.86***	−0.54†	0.05	−0.02	0.35*	0.05	none
**Bacteroidetes, Cecum-RT**	2.61^a^ (0.062)†	0.31	−0.36*	−0.60*	0.27	−0.08	0.29*	0.08	none
**Firmicutes, Ileum-RT**	2.50^b^ (0.068)†	0.33	0.40	−0.57†	0.44	−0.01	0.10	0.10	MGAM (−0.52)*
**Firmicutes, Cecum-RT**	6.98^c^ (0.001)**	0.69	1.29***	−0.99**	0.86**	0.18†	0.06	0.40*	MGAM (−0.50)*, GLUT2 (−0.46)*
**Firm./Bac., Ileum-RT**	3.43^b^ (0.024)*	0.45	1.43**	−0.19	0.19	0.04	−0.27	0.48†	GLUT2 (−0.61)*
**Firm./Bac., Cecum-RT**	5.13^b^ (0.005)**	0.58	1.47***	0.27	0.21	0.19	−0.22	−0.02	SI (−0.93)**
**Proteobacteria, Ileum-454**	2.47^b^ (0.071)†	0.33	−1.05	2.76**	−2.31*	0.01	−0.79†	−0.59†	MGAM (1.21)†
**Proteobacteria, Cecum-454**	5.41^b^ (0.004)**	0.59	−1.21	3.34***	−3.56***	−0.03	−0.68†	−0.38	MGAM (1.59)**
**BetaProteobacteria, Ileum-454**	1.14^a^ (0.385)	0.04	−0.14	0.61	−0.87	0.05	−0.26	−0.16	none
**BetaProteobacteria, Cecum-454**	5.64^a^ (0.003)**	0.57	−0.16	1.43*	−2.07**	0.27	−0.44	0.08	none

a - on 6 and 15 degrees of freedom.

b - on 7 and 14 degrees of freedom.

c - on 8 and 13 degrees of freedom.

STDev - Change in log-transformed outcome variable levels per unit standard deviation increase in predictor variable (main effect variables or interaction terms).

*, p<0.05;

**, p<0.01;

***, p<0.001;

†, p<0.1 (trend).

Firmicute levels significantly decreased as SI levels increased in cecum. Cecal Firmicute levels were increased as the levels of MGAM and GLUT2 increased. The levels of Firmicutes in the cecum were higher in AUT-GI compared to Control-GI children after adjusting for the expression of disaccharidases and transporters. Significant interaction was found between diagnostic status and MGAM and GLUT2 levels in the Firmicute models. Whereas higher levels of MGAM and GLUT2 were associated with higher levels of Firmicutes among Control-GI children, the effects of MGAM and GLUT2 on Firmicutes were not present in AUT-GI children.

Disaccharidases and transporter levels were not significant predictors of the ratios of Firmicutes to Bacteroidetes in ileum or cecum. However, the interaction terms with GLUT2 in the ileum and SI in the cecum were significant.

Proteobacteria abundance significantly increased as the levels of SI increased, but decreased as MGAM increased for both ileum and cecum. However, the interaction terms with MGAM in both ileum and cecum were significant, implying that the magnitude of decline is significantly smaller among AUT-GI children. Betaproteobacteria abundance was positively associated with SI and inversely associated with MGAM only in cecum; none of the interactions were significant. In addition, Proteobacteria and Betaproteobacteria abundance were not significantly different between AUT-GI and Control-GI children after adjusting for the expression of all disaccharidases and transporters. Overall, these results suggest that expression levels of disaccharidases and transporters are associated with the abundance of Bacteroidetes, Firmicutes, and Betaproteobacteria in the mucoepithelium.

The levels of Betaproteobacteria in the ileum and cecum were higher in AUT-GI children with deficiencies in all 5 disaccharidases and transporters versus AUT-GI children with fewer than 5 disaccharidase and transporter deficiencies (**Figure S8A, B**). Levels of CDX2 were lower in AUT-GI children with levels of Betaproteobacteria above the 75^th^ percentile of Control-GI children compared to AUT-GI children with levels of Betaproteobacteria below the 75^th^ percentile of Control-GI children (**Figure S8C, D**). These results suggest a potential link between increased levels of Betaproteobacteria, reduced levels of CDX2 expression, and overall deficiencies in disaccharidases and transporters.

### Timing of GI disturbances relative to onset of autism is associated with changes in Clostridiales members

In this cohort, the onset of GI symptoms was reported to occur before or at the same time as the development of autism in 67% of AUT-GI children. As a sub-analysis, we sought to determine whether the timing of GI onset relative to autism onset was associated with gene expression and bacterial variables.

**Figure 6:**
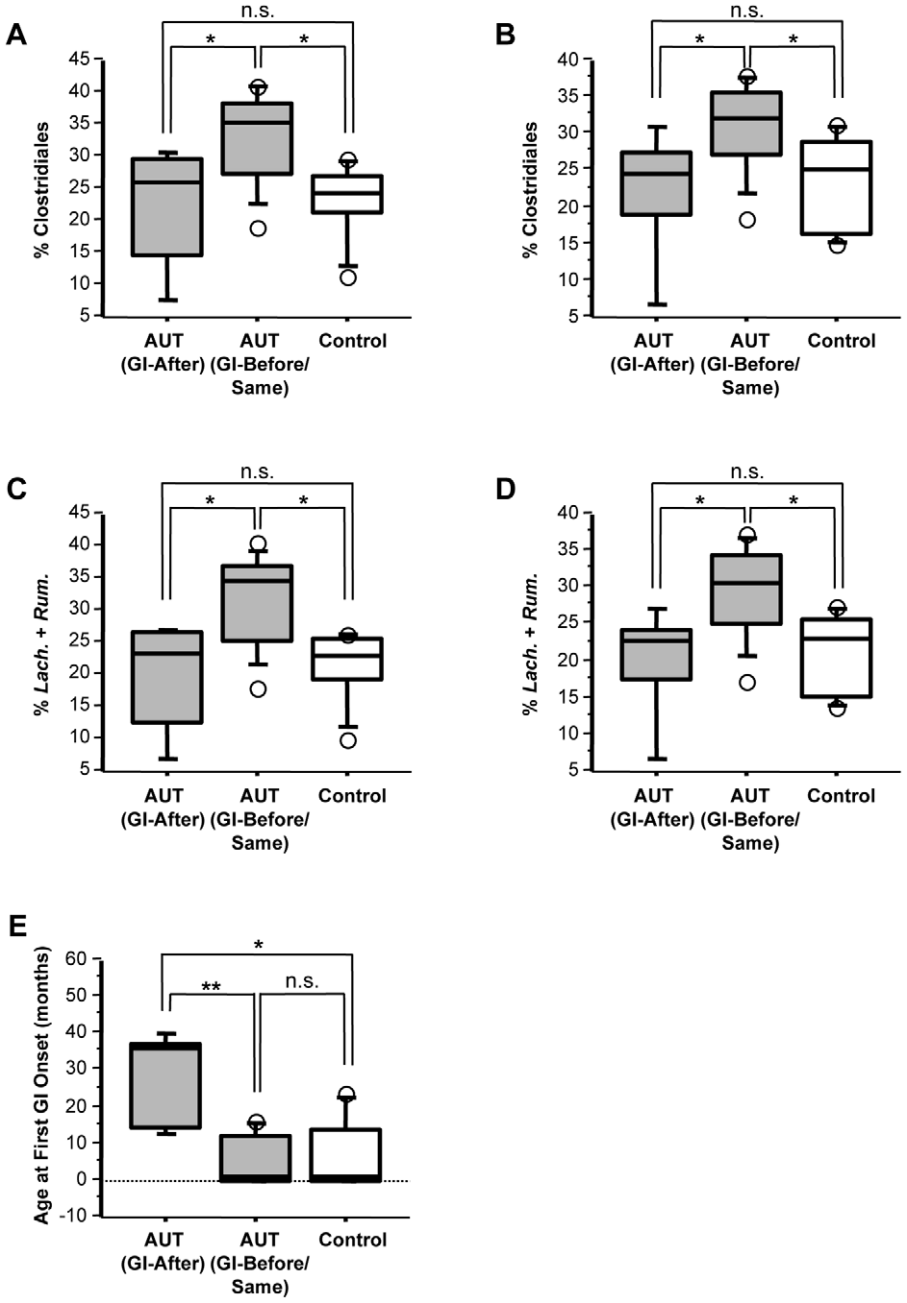
Levels of Clostridiales members in AUT-GI patients stratified by timing of GI onset. (**A–B**) Abundance of Clostridiales from ileal (**A**) and cecal (**B**) biopsies from AUT-GI and Control-GI patients (n = 7), with AUT-GI stratified by whether the onset of GI symptoms occurred after (n = 5) the onset of autism symptoms (GI-After) or before and at the same time (n = 10) as autism symptoms (GI-Before/Same). [**A**: AUT (GI-After) vs. AUT (GI-Before/Same), Mann-Whitney, p = 0.028; AUT (GI-Before/Same) vs. Control-GI, Mann-Whitney, p = 0.015; AUT (GI-After) vs. Control-GI, Mann-Whitney, p = 0.935] [**B**: AUT (GI-After) vs. AUT (GI-Before/Same), Mann-Whitney, p = 0.037; AUT (GI-Before/Same) vs. Control-GI, Mann-Whitney, p = 0.019; AUT (GI-After) vs. Control-GI, Mann-Whitney, p = 0.935]. (**C–D**) Cumulative abundance of *Lachnospiraceae* and *Ruminococcaceae* from ileal (**C**) and cecal (**D**) biopsies from AUT-GI and Control-GI patients (n = 7), with AUT-GI stratified by whether the onset of GI symptoms occurred after (n = 5) the onset of autism symptoms or before and at the same time (n = 10) as autism symptoms [**C**: AUT (GI-After) vs. AUT (GI-Before/Same), Mann-Whitney, p = 0.028; AUT (GI-Before/Same) vs. Control-GI, Mann-Whitney, p = 0.015; AUT (GI-After) vs. Control-GI, Mann-Whitney, p = 0.808] [**D**: AUT (GI-After) vs. AUT (GI-Before/Same), Mann-Whitney, p = 0.020; AUT (GI-Before/Same) vs. Control-GI, Mann-Whitney, p = 0.011; AUT (GI-After) vs. Control-GI, Mann-Whitney, p = 0.685]. (**E**) Age at GI onset (in months) for AUT-GI and Control-GI patients, with AUT-GI stratified by whether GI onset occurred after (n = 5) the onset of autism symptoms or before and at the same time (n = 10) as autism symptoms [**E**: AUT (GI-After) vs. AUT (GI-Before/Same), Mann-Whitney, tied p = 0.007; AUT (GI-Before/Same) vs. Control-GI, Mann-Whitney, tied p = 0.757; AUT (GI-After) vs. Control-GI, Mann-Whitney, tied p = 0.027]. *, p<0.05; **, *p*<0.01; n.s., not significant.

Patients were stratified based on whether the first episode of GI symptoms occurred before or at the same time (within the same month) as the onset of autism (AUT-GI-Before or Same group) or whether the first episode of GI symptoms occurred after the onset of autism (AUT-GI-After group). The timing of GI onset was not associated with levels of disaccharidase, hexose transporter or CDX2 transcripts, Bacteroidetes, Proteobacteria or Betaproteobacteria (data not shown). However, a significant effect was observed for the levels of Clostridiales and cumulative levels of *Lachnospiraceae* and *Ruminococcaceae* in both the ileum and cecum (**Figure 6A–D**). Whereas only a trend toward increased Clostridiales and cumulative levels of *Lachnospiraceae* and *Ruminococcaceae* were observed when comparing all AUT-GI and Control-GI children (**Figure S5G, H** and **Figure 4G, H**), stratification based on timing of GI onset revealed a significant increase in these variables in both the ileum and cecum of the AUT-GI-Before or Same group relative to all Control-GI children (**Figure 6A**: Clostridiales-ileum, Mann-Whitney, p = 0.015; **Figure 6B**: Clostridiales-cecum, Mann-Whitney, p = 0.019; **Figure 6C**: *Lach.*+*Rum.*-ileum, Mann-Whitney, p = 0.015; **Figure 6D**: *Lach.*+*Rum.*-cecum, Mann-Whitney, p = 0.011). Furthermore, the levels of Clostridiales and cumulative levels of *Lachnospiraceae* and *Ruminococcaceae* were significantly higher in the AUT-GI-Before or Same group relative to the AUT-GI-After group (**Figure 6A**: Clostridiales-ileum, Mann-Whitney, p = 0.028; **Figure 6B**: Clostridiales-cecum, Mann-Whitney, p = 0.037; **Figure 6C**: *Lach.*+*Rum.*-ileum, Mann-Whitney, p = 0.028; **Figure 6D**: *Lach.*+*Rum.*-cecum, Mann-Whitney, p = 0.020); the AUT-GI-After group was not significantly different from the Control-GI group. As expected, the AUT-GI-After group had a significantly older age at first onset of GI symptoms [median age in months, (interquartile range, IQR) = 36, (22.5)] compared to the AUT-GI-Before or Same group [median age in months, (interquartile range, IQR) = 1, (12)] (**Figure 6E**: Mann-Whitney, p = 0.007), and was also higher than the Control-GI group [median age in months, (interquartile range, IQR) = 1, (14)] (**Figure 6E**: Mann-Whitney, p = 0.027). The age at first GI onset was not significantly different between the AUT-GI-Before or Same group and the Control-GI group (**Figure 6E**: Mann-Whitney, p = 0.757). Thus, the increased levels of Clostridiales in the AUT-GI-Before or Same group as compared to the Control-GI group were not influenced by differences in age of onset of GI symptoms between these two groups. These results suggest that the timing of onset of GI symptoms relative to onset of autism or the age at first GI onset may be associated with increases in Clostridiales.

### Associations between gene expression, bacterial abundance, and food allergies and other comorbid atopic manifestations

A National Survey of Children's Health, performed under the auspices of the Centers for Disease Control, reported that parents of autistic children reported more allergy symptoms than control children, and food allergies were the most prevalent complaint [39]. Parental reports of food allergies (FA) in our cohort were reported with similar frequency in AUT-GI (67%) and Control-GI (71%) children. Milk-related (MA) and wheat-related (WA) allergies were the most commonly reported allergies in both groups (**Table 1** and **Table S1**). To determine whether FA was associated with gene expression or bacterial levels, patients in the AUT-GI group and Control-GI group were stratified based on reports of any FA (**Table 5**), MA (**Table 6**), or WA (**Table 7**).

**Table 5:** Association of any food allergy (FA) with host gene expression and bacterial phylotypes in AUT-GI children.

	Food Allergies (FA)	
Variable	AUT(+FA) vs. Control(+FA)^a^, p-value^MW^, [effect in AUT(+FA)]	AUT(−FA) vs. AUT(+FA)^b^, p-value^MW^, [effect in AUT(+FA)]
**GLUT2**	0.014*, [decreased]	0.037*, [decreased]
**Bacteroidetes IL(RT)**	0.002**, [decreased]	0.806, [no change]
**Bacteroidetes CEC(RT)**	0.005**, [decreased]	0.713, [no change]
**Bacteroidetes IL(454)**	0.037*, [decreased]	0.221, [no change]
**Bacteroidetes CEC(454)**	0.050*, [decreased]	0.713, [no change]
**Firmicutes IL(RT)**	0.221, [no change]	0.037*, [increased]
**Firmicutes CEC(RT)**	0.037*, [increased]	0.010*, [increased]
**Firm./Bacteroid. Ratio IL(RT)**	0.003**, [increased]	0.037*, [increased]
**Firm./Bacteroid. Ratio CEC(RT)**	0.005**, [increased]	0.020*, [increased]
**Beta-proteobacteria IL(454)**	0.050†, [increased]	0.066†, [increased]
**Beta-proteobacteria CEC(454)**	0.028*, [increased]	0.037*, [increased]

a -AUT(+FA), n = 10; Control(+FA), n = 5.

b -AUT(−FA), n = 5; AUT(+FA), n = 10.

MW - Mann-Whitney test.

*, p<0.05;

**, p<0.01;

†, p<0.1 (trend).

**Table 6:** Association of milk allergy (MA) with host gene expression and bacterial phylotypes in AUT-GI children.

	Milk Allergies (MA)	
Variable	AUT(+MA) vs. Control(+MA)^a^, p-value^MW^, [effect in AUT(+MA)]	AUT(−MA) vs. AUT(+MA)^b^, p-value^MW^, [effect in AUT(+MA)]
**SI**	0.006**, [decreased]	0.099†, [decreased]
**MGAM**	0.006**, [decreased]	0.045*, [decreased]
**GLUT2**	0.009**, [decreased]	0.013*, [decreased]
**CDX2**	0.072†, [decreased]	0.034*, [decreased]
**Bacteroidetes IL(RT)**	0.003**, [decreased]	0.480, [no change]
**Bacteroidetes CEC(RT)**	0.003**, [decreased]	0.289, [no change]
**Bacteroidetes IL(454)**	0.028*, [decreased]	0.637, [no change]
**Bacteroidetes CEC(454)**	0.020*, [decreased]	0.637, [no change]
**Firmicutes IL(RT)**	0.205, [no change]	0.059†, [increased]
**Firmicutes CEC(RT)**	0.053†, [increased]	0.099†, [increased]
**Firm./Bacteroid. Ratio IL(RT)**	0.004**, [increased]	0.034*, [increased]
**Firm./Bacteroid. Ratio CEC(RT)**	0.006**, [increased]	0.045*, [increased]
**Beta-proteobacteria IL(454)**	0.020*, [increased]	0.013*, [increased]
**Beta-proteobacteria CEC(454)**	0.009**, [increased]	0.007**, [increased]

a -AUT(+MA), n = 9; Control(+MA), n = 5.

b -AUT(−MA), n = 6; AUT(+MA), n = 9.

MW - Mann-Whitney test.

*, p<0.05;

**, p<0.01;

†, p<0.1 (trend).

**Table 7:** Association of wheat allergy (WA) with host gene expression and bacterial phylotypes in AUT-GI children.

	Wheat Allergies (WA)	
Variable	AUT(+WA) vs. Control(+WA)^a^, p-value^MW^, [effect in AUT(+WA)]	AUT(−WA) vs. AUT(+WA)^b^, p-value^MW^, [effect in AUT(+WA)]
**Bacteroidetes IL(RT)**	0.007**, [decreased]	0.643, [no change]
**Bacteroidetes CEC(RT)**	0.017*, [decreased]	0.643, [no change]
**Bacteroidetes IL(454)**	0.017*, [decreased]	0.488, [no change]
**Bacteroidetes CEC(454)**	0.089†, [decreased]	0.908, [no change]
**Firmicutes IL(RT)**	0.174, [no change]	0.083†, [increased]
**Firmicutes CEC(RT)**	0.089†, [increased]	0.008**, [increased]
**Firm./Bacteroid. Ratio IL(RT)**	0.011*, [increased]	0.203, [no change]
**Firm./Bacteroid. Ratio CEC(RT)**	0.011*, [increased]	0.049*, [increased]
**Beta-proteobacteria IL(454)**	0.089†, [increased]	0.643, [no change]
**Beta-proteobacteria CEC(454)**	0.042*, [increased]	0.418, [no change]

a -AUT(+WA), n = 8; Control(+WA), n = 4.

b -AUT(−WA), n = 7; AUT(+WA), n = 8.

MW - Mann-Whitney test.

*, p<0.05;

**, p<0.01;

†, p<0.1 (trend).

Stratification by any FA revealed a significant effect for levels of GLUT2, ileal and cecal Firmicutes, ileal and cecal ratios of Firmicutes to Bacteroidetes, and cecal Betaproteobacteria (**Table 5**). No effect was observed for the levels of Bacteroidetes, which were significantly reduced in AUT-GI children independent of FA status.

Stratification by MA status revealed even more significant effects (**Table 6**). Significant effects were observed for MGAM, GLUT2, and CDX2 expression, as well as ileal and cecal ratios of Firmicutes to Bacteroidetes, and ileal and cecal Beta-proteobacteria. Additional trends were observed for SI expression and ileal and cecal Firmicutes. No effect was observed for the levels of Bacteroidetes, which were significantly reduced in AUT-GI children independent of MA status.

Stratification by WA status was associated with a significant effect only for cecal levels of Firmicutes, though this effect was highly significant [AUT(+WA) vs. AUT(−WA): Mann-Whitney, p-value = 0.008], and the cecal ratio of Firmicutes to Bacteroidetes (**Table 7**).

These results suggest that changes in the expression of some disaccharidases and transporters and CDX2, as well as changes in the abundance of some bacterial phylotypes, are significantly associated with reported FA, especially MA. Whereas the levels of Firmicutes, the ratio of Firmicutes to Bacteroidetes, and levels of Betaproteobacteria were increased in AUT-GI children with FA, the levels of Bacteroidetes were not significantly different. This suggests that the levels of Bacteroidetes were significantly decreased in AUT-GI children, independent of FA status.

Atopic disease manifestations (AD: asthma, allergic rhinitis, or atopic dermatitis) were the most commonly reported comorbid conditions in both AUT-GI and Control-GI children. The frequency of AD tended to be higher in the Control-GI group (100%) than in the AUT-GI group (53%) (**Table 1**: Fisher's Exact Test, 2-sided p = 0.051). In the combined group (all AUT-GI and Control-GI patients), 86.7% of children with reported FA had at least one reported AD; only 28.6% of children without reported FA had one or more AD (Fisher's Exact Test, 2-sided p = 0.014). As AD was associated with reported FA, we sought to determine whether AD manifestation was also associated with changes in disaccharidases and transporters or bacterial parameters. Stratification of subjects by AD status revealed that cecal Firmicutes and the cecal ratio of Firmicutes to Bacteroidetes were higher in AUT-GI children with AD compared to Control-GI children with AD [**Table 8**: AUT(+AD) vs. Control(+AD); Firmicutes CEC(RT), Mann-Whitney, p = 0.015; Firm./Bacteroid. Ratio CEC(RT), Mann-Whitney, p = 0.002] and AUT-GI children without AD [**Table 8**: AUT(−AD) vs. AUT(+AD); Firmicutes CEC(RT), Mann-Whitney, p = 0.049; Firm./Bacteroid. Ratio CEC(RT), Mann-Whitney, p = 0.049].

**Table 8:** Association of atopic disease (AD) status with host gene expression and bacterial phylotypes in AUT-GI children.

	Atopic Disease (AD)	
Variable	AUT(+AD) vs. Control(+AD)^a^, p-value^MW^, [effect in AUT(+AD)]	AUT(−AD) vs. AUT(+AD)^b^, p-value^MW^, [effect in AUT(+AD)]
**Bacteroidetes IL(RT)**	0.008**, [decreased]	0.563, [no change]
**Bacteroidetes CEC(RT)**	0.028*, [decreased]	0.418, [no change]
**Bacteroidetes IL(454)**	0.049*, [decreased]	0.643, [no change]
**Bacteroidetes CEC(454)**	0.064†, [decreased]	0.908, [no change]
**Firmicutes IL(RT)**	0.064†, [increased]	0.133, [no change]
**Firmicutes CEC(RT)**	0.015*, [increased]	0.049*, [increased]
**Firm./Bacteroid. Ratio IL(RT)**	0.002**, [increased]	0.064†, [increased]
**Firm./Bacteroid. Ratio CEC(RT)**	0.006**, [increased]	0.049*, [increased]
**Beta-proteobacteria IL(454)**	0.049*, [increased]	0.203, [no change]
**Beta-proteobacteria CEC(454)**	0.028*, [increased]	0.133, [no change]

a -AUT(+AD), n = 8; Control(+AD), n = 7.

b -AUT(−AD), n = 7; AUT(+AD), n = 8.

MW - Mann-Whitney test.

* , p<0.05;

** , p<0.01;

† , p<0.1 (trend).

## Discussion

**Figure 7:**
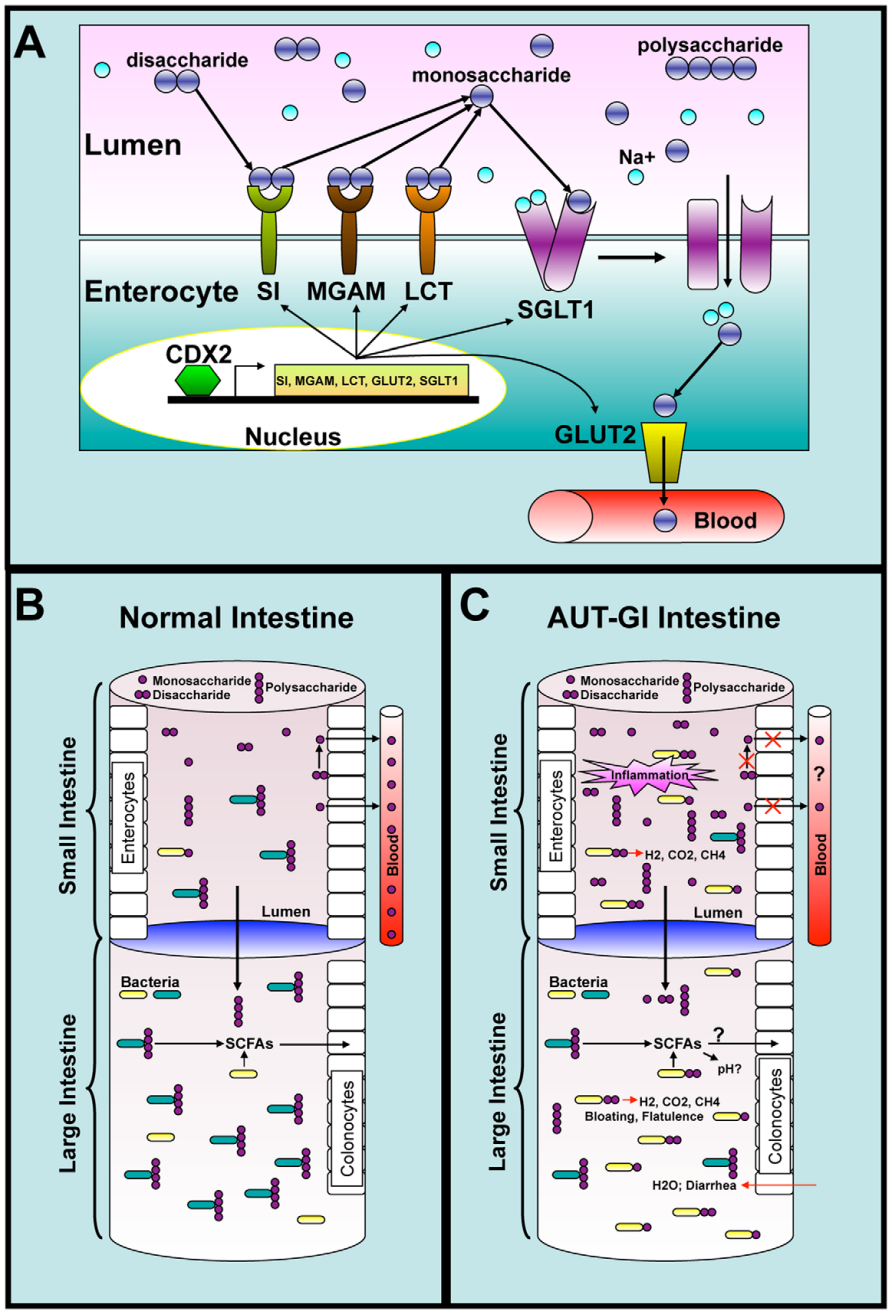
A model for GI disease in children with autism. **A**) Schematic representation of enterocyte-mediated digestion of disaccharides and transport of monosaccharides in the small intestine. Disaccharidases (SI, MGAM, and LCT) in the enterocyte brush border break down disaccharides into their component monosaccharides. The monosaccharides, glucose and galactose, are transported from the small intestinal lumen into enterocytes by the sodium-dependent transporter SGLT1. On the basolateral enterocyte membrane, GLUT2, transports glucose, galactose, and fructose out of enterocytes and into the circulation. The expression levels of disaccharidases and hexose transporters may be controlled, in part, by the transcription factor CDX2. **B**) In the normal small intestine, where expression of disaccharidases and hexose transporters are high, nearly all disaccharides are efficiently digested and monosaccharides are absorbed from the lumen. **C**) In the AUT-GI intestine, where expression of disaccharidases and hexose transporters are deficient, mono- and disaccharides accumulate in the lumen of the ileum and cecum resulting in dysbiosis, diarrhea, bloating, and flatulence.

Although the major deficits in ASD are social and cognitive, many affected individuals with ASD also have substantial GI morbidity. Major findings in this study that may shed light on GI morbidity in ASD include the observations that: (1) levels of transcripts for disaccharidases and hexose transporters are reduced in AUT-GI children; (2) AUT-GI children have microbial dysbiosis in the mucoepithelium; and (3) dysbiosis is associated with deficiencies in host disacharidase and hexose transporter mRNA expression. Based on these findings, we propose a model whereby deficiencies in disaccharidases and hexose transporters alter the milieu of carbohydrates in the distal small intestine (ileum) and proximal large intestine (cecum), resulting in the supply of additional growth substrates for bacteria. These changes manifest in significant and specific compositional changes in the microbiota of AUT-GI children (see **Figure 7A–C**).

A previous report on GI disturbances in ASD found low activities of at least one disaccharidase or glucoamylase in duodenum in 58% of children [7]. In our study, 93.3% of AUT-GI children had decreased mRNA levels for at least one of the three disaccharidases (SI, MGAM, or LCT). In addition, we found decreased levels of mRNA for two important hexose transporters, SGLT1 and GLUT2. Congenital defects in these enzymes and transporters are extremely rare [40], [41], and even the common variant for adult-type hypolactasia was not responsible for reduced LCT expression in AUT-GI children in this cohort. Therefore, it is unlikely that the combined deficiency of disaccharidases (maldigestion) and transporters (malabsorption) are indicative of a primary malabsorption resulting from multiple congenital or acquired defects in each of these genes. Transcripts for the enterocyte marker, villin, were not reduced in AUT-GI ilea and did not predict the expression levels of any of the disaccharidases or transporters in multiple regression models. This suggests that a general loss of enterocytes is unlikely. However, we cannot exclude the possibility that defects in the maturational status of enterocytes or enterocyte migration along the crypt-villus axis contribute to deficits in disaccharidase and transporter expression [42].

The ileal expression of CDX2, a master transcriptional regulator in the intestine, was a significant predictor of mRNA expression of all five disaccharidases and transporters in AUT-GI and Control-GI children, based on linear regression models. However, as ASD status remained a significant predictor of disaccharidase mRNA expression even after adjusting for CDX2 and villin, additional factors must also contribute. One potential factor is diet. Dietary intake of carbohydrates can regulate the mRNA expression of disaccharidases and hexose transporters in mice and rats [43], [44], [45]. Several studies suggest that ASD children exhibit feeding selectivity and aberrant nutrient consumption [46], [47], [48], [49], [50], [51], [52]. However, of the four studies reporting on carbohydrate intake, none found differences in total carbohydrate intake in ASD children [47], [48], [49], [50]. Furthermore, one study found no association between dietary intake of macronutrients (i.e., carbohydrates, proteins, or fats) and GI symptoms [47]. Unfortunately, dietary diaries for the period immediately preceding biopsy were not available for the children evaluated in our study; hence, the extent to which dietary intake affected intestinal gene expression could not be determined.

Hormonal and growth factor regulation of some disaccharidases and hexose transporters have been reported in *in vitro* studies and in animals [53], [54]. Inflammatory cytokines can regulate SI gene expression in human intestinal epithelial cells *in vitro* [55]. Thus, immunological or hormonal imbalances reported in ASD children [5], [8], [9], [10], [11], [12], [56], [57], [58] may also contribute to expression deficits. Additionally, intestinal microbes can influence the expression of disaccharidases and transporters [59] through the influence of pathogen-associated molecular patterns (PAMPs) and butyrate (a byproduct of bacterial fermentation) on CDX2 expression and activity [60], [61], [62], [63]. In this regard, the observation that CDX2 was decreased in AUT-GI children with increased levels of Betaproteobacteria may be important.

Whatever the underlying mechanisms, reduced capacity for digestion and transport of carbohydrates can have profound effects. Within the intestine, malabsorbed carbohydrates can lead to osmotic diarrhea [64]; non-absorbed sugars may also serve as substrates for intestinal microflora that produce fatty acids and gases (methane, hydrogen, and carbon dioxide), promoting additional GI symptoms such as bloating and flatulence [65]. The deficiency of even a single gene in this important pathway can result in severe GI disease, as occurs with glucose-galactose malabsorption syndrome caused by SGLT1 deficiency, Fanconi-Bickel syndrome resulting from GLUT2 mutations, sucrase-isomaltase deficiency, and congenital lactase deficiency [40], [41].

Changes in the type and quantity of dietary carbohydrates can influence composition and function of intestinal microflora [66], [67], [68]; thus, we reasoned that carbohydrate maldigestion and malabsorption, resulting from deficient expression of disaccharidases and hexose transporters, might have similar effects in AUT-GI subjects. Pyrosequencing analysis of mucoepithelial bacteria revealed significant multicomponent dysbiosis in AUT-GI children, including decreased levels of Bacteroidetes, an increase in the Firmicute/Bacteroidete ratio, increased cumulative levels of Firmicutes and Proteobacteria, and increased levels of bacteria in the class Betaproteobacteria.

A recent pyrosequencing study reported an increase in Bacteroidetes in fecal samples of ASD subjects [69]. Although these findings may appear to be incongruent with those reported here, our data were obtained using biopsies rather than free fecal material. Others have reported differences in the composition of fecal versus mucosal microflora [35], [70], [71], [72]. Only about 50% of cells in feces are viable, with dead and injured cells making up the remaining fractions [73]. The loss of Bacteroidetes from the mucoepithelium as a result of death, injury, or competition for binding in the mucosal space can result in increased wash out of Bacteroidete cells into the fecal stream. Thus, higher levels of Bacteroidetes in feces could be indicative of an inability to thrive in the mucosal microbiome rather than an indication that Bacteroidetes are found at higher levels in the microbiome.

The trend toward increased Firmicutes was largely attributable to Clostridia with *Ruminococcaceae* and *Lachnospiraceae* as major contributors. Several *Ruminococcaceae* and *Lachnospiraceae* are known butyrate producers and may thus influence short-chain fatty acid (SCFA) levels [74]. SCFA influence colonic pH, and some *Bacteroides sp.* are sensitive to acidic pH [75]. Three previous reports indicated differences in Clostridia species in children with ASD, including greater abundance of *Clostridium* clusters I, II, XI and *C. bolteae* [14], [15], [16]. Stratification of AUT-GI children based on the timing of GI symptom development relative to autism onset revealed that the levels of Clostridiales and cumulative levels of *Lachnospiraceae* and *Ruminococcaceae* were significantly higher in AUT-GI children for whom GI symptoms developed before or at the same time as the onset of autism symptoms compared to AUT-GI children for whom GI symptoms developed after the onset of autism and compared to Control-GI children. However, we cannot discern whether changes in Clostridiales occurred before the onset of autism in this subgroup. We can only conclude that increased levels of Clostridiales members in biopsies taken after the development of both GI symptoms and autism are associated with the timing of GI onset relative to autism onset in this cohort. Although the reason for this association remains unclear, this finding may suggest that the timing of GI onset relative to autism is an important variable to consider in the design of future prospective studies investigating the microbiota of children with autism.

Although we found only a trend for increased Firmicutes in AUT-GI children, the cumulative levels of Firmicutes and Proteobacteria were significantly higher. These results suggest that, in some patients, a decrease in Bacteroidetes is associated with an increase in Firmicutes, whereas, in others, increases in Proteobacteria are associated with a reduced abundance of Bacteroidetes. Three AUT-GI patients had high levels of Alpha-, Beta-, or Gammaproteobacteria. In addition, the AUT-GI group had elevated levels of Betaproteobacteria compared to the Control-GI group, primarily reflecting the presence of *Alcaligenaceae*. *Alcaligenaceae* sequences were not detected in any tissues from Control-GI children.

Deficient digestion and absorption of di- and monosaccharides in the small intestine may alter the balance of growth substrates, thus eliminating the growth advantages that Bacteroidetes enjoy in the healthy intestine and enabling competitive growth of bacterial phylotypes better suited for growth on undigested and unabsorbed carbohydrates. In support of this hypothesis, multiple linear regression models demonstrated that levels of ileal SGLT1 and SI mRNA were associated with levels of Bacteroidetes in ileum and cecum, or cecum alone, respectively. Levels of ileal SI, MGAM, and GLUT2 mRNA were associated with levels of cecal Firmicutes, although the magnitude of the effects of MGAM and GLUT2 differed between AUT-GI and Control-GI children. Significant associations were also observed between levels of SI and MGAM mRNA and of Proteobacteria in ileum and cecum, and of Betaproteobacteria in cecum. Although deficiencies in disaccharidase and transporter expression appear to at least partially contribute to these alterations in the AUT-GI microbiota, diagnostic status remained a significant predictor of Bacteroidete and cecal Firmicute abundance even after adjusting for gene expression.

Metabolic interactions between intestinal microflora and their hosts are only beginning to be understood. Nonetheless, there is already abundant evidence that microflora can have system-wide effects [76], [77], [78], [79], [80], [81], [82], [83] and influence immune responses, brain development and behavior [24], [25], [26], [84], [85]. We acknowledge that this is a small study comprising 22 subjects. The small sample size evaluated in this study is a limitation arising from the difficulty in obtaining biopsies from young children undergoing invasive endoscopic examination. This caveat notwithstanding, our data show that at least some children with autism have a distinct intestinal profile that is linked to deficient expression of disaccharidases and hexose transporters, potentially promoting maldigestion, malabsorption, and multicomponent, compositional dysbiosis. Although the underlying cause of these changes and the extra-intestinal effects these changes may elicit remain speculative, the identification of specific molecular and microbial signatures that define GI pathophysiology in AUT-GI children sets the stage for further research aimed at defining the epidemiology, diagnosis, and informed treatment of GI symptoms in autism.

## Materials and Methods

### Ethics Statement

The Institutional Review Board (IRB) at Columbia University Medical Center reviewed and approved the use of de-identified residual ileal and cecal samples, obtained as described in an earlier publication [86], and waived the need for patient consent for these analyses, as all samples were analyzed anonymously. Samples assessed in the present study were restricted to those derived from male children from the original cohort between 3 and 5 years of age to control for confounding effects of gender and age on intestinal gene expression and the microbiota. This subset comprised 15 AUT-GI (patients #1-15) and 7 Control-GI (patients #16-22) patients.

### Clinical Procedures

Specific clinical procedures for defining neuropsychiatric and regression status in this cohort have been previously described [86]. Briefly, neuropsychiatric status was established for all subjects using Diagnostic and Statistical Manual-Fourth Edition, Text Revision (DSM-IV-TR) diagnostic criteria. Only cases meeting full DSM-IV-TR criteria for Autistic Disorder (AUT) were included for further analysis. DSM-IV-TR diagnosis of AUT was confirmed by certified raters using the Autism Diagnostic Interview-Revised (ADI-R). Regression status was determined based on ADI-R and Shortened CPEA Regression Interview. Control-GI children were evaluated in the same manner as cases to exclude subjects with any developmental disturbances, including ASD. Age of AUT onset was determined by an ADI-R certified interviewer. Questions posed to parents in standardized data collection forms regarding GI symptoms were based on previous work [27]. Symptoms were only reported if the child had experienced the specific GI symptoms, including food allergies, for 3 consecutive months. History of medication use, presence of comorbid conditions, age at first GI episode, and presence and type of food allergies were also acquired through parental questionnaires.

### RNA and DNA extraction

All biopsies were snap frozen at collection and stored at −80°C until extraction. RNA and DNA were extracted sequentially from individual ileal and cecal biopsies [total of 176 biopsies from 15 AUT-GI patients and 7 Control-GI patients: 8 biopsies per patient (4 each from ileum and cecum), yielding 88 ileal and 88 cecal biopsies] in TRIzol (Invitrogen) using standard protocols. RNA from half of the biopsies (2 ileal and 2 cecal biopsies per AUT-GI or Control-GI patient) was derived from residual extracts from the original study completed in 2008 [86]. RNA from the other half of the biopsies (the remaining 2 ileal and 2 cecal biopsies per AUT-GI or Control-GI patient) was newly extracted from stored biopsies (stored undisturbed at −80°C) at the inception of the current study in 2008. The interphase and organic phase fractions were stored at −80°C, following RNA extraction, for subsequent DNA extraction. All extractions were stored in aliquots at −80°C to avoid repeated freeze thawing of samples. RNA and DNA concentrations, purity, and integrity were determined for all residual extracts and newly extracted biopsies just prior to cDNA synthesis for mRNA expression studies and just prior to PCR of newly extracted DNA using a Nanodrop ND-1000 Spectrophotometer (Nanodrop Technologies) and Bioanalyzer (Agilent Technologies).

### Quantitative Real-Time PCR of human mRNA

Intron/exon spanning, gene-specific PCR primers and probes (**Table S6**) for SI, MGAM, LCT, SGLTI, GLUT2, villin, and CDX2, with GAPDH and β-actin as dual housekeeping gene controls, were designed for real-time PCR using Primer Express 1.0 software (Applied Biosystems). Taqman probes were labeled with the reporter FAM (6-carboxyfluorescein) and the quencher BBQ (Blackberry) (TIB MolBiol). Assays were designed and implemented as previously described [87], [88], [89]. For more details, see **Text S2**. Levels of mRNA expression for each gene and in each AUT-GI individual were considered significantly increased or decreased if they were above the 75^th^ percentile or below the 25^th^ percentile, respectively, of gene expression obtained for all Control-GI children and were at least two-fold increased or decreased from the Control-GI mean (**Figure 2A** and **Table S3**).

### Lactase genotyping

Genomic DNA from AUT-GI and Control-GI patients was subjected to previously-described, PCR-restriction fragment length polymorphism (PCR-RFLP) analysis for the C/T-13910 and G/A-22018 polymorphisms associated with adult-type hypolactasia with minor modifications [90]. For details, see **Text S1**, **Text S2**, and **Figure S1**.

### Barcoded pyrosequencing of intestinal microbiota

PCR was carried out using bacterial 16S rRNA gene-specific (V2-region), barcoded primers as previously described [91]. Barcoded 16S rRNA genes were amplified from DNA samples from the 88 ileal biopsies and 88 cecal biopsies. Amplicons were sequenced at 454 Life Sciences on a GS FLX sequencer. For primer sequences and detailed methods, see **Text S2**.

### Quantitative Real-time PCR of Bacteroidete and Firmicute 16S rRNA genes

Primer sequences and PCR conditions used for bacterial real-time PCR assays to quantify Bacteroidetes, Firmicutes, and total Bacterial 16S rRNA genes have been previously described [92], [93], and details are outlined in the **Text S2**; primer sequences are listed in **Table S6**. Copy numbers of Bacteroidetes, Firmicutes, or Firmicute to Bacteroidete ratios that were above the 75^th^ percentile or below the 25^th^ percentile of Control-GI children were scored as an increase or decrease, respectively (**Figure 2B, C**). Percent changes in bacterial parameters for individuals in the AUT-GI group were determined based on the mean levels in Control-GI children (**Table S4**).

### Bioinformatic analysis of pyrosequencing reads

Pyrosequencing reads ranging from 235 to 300 base pairs in length (encompassing all sequences within the major peak obtained from pyrosequencing) were filtered for analysis. Low-quality sequences - i.e., those with average quality scores below 25 - were removed based on previously described criteria [91], [94]. Additionally, reads with any ambiguous characters were omitted from analysis. Sequences were then binned according to barcode, followed by removal of primer and barcode sequences. Taxonomic classifications of bacterial 16S rRNA sequences were obtained using the RDP classifier tool (http://rdp.cme.msu.edu/) with a minimum 80% bootstrap confidence estimate. To normalize data for differences in total sequences obtained per patient, phylotype abundance was expressed as a percentage of total bacterial sequence reads per patient at all taxonomic levels. Taxonomy note: the RDP classifier binned all of the limited number of sequences obtained for the phylum Cyanobacteria into the chloroplast-derived genus *Streptophyta*. Heatmaps were constructed with MeV (Version 4.5.0), using abundance data from pyrosequencing reads. Heatmap scales were made linear where possible, with the upper limit reflecting the highest abundance recorded for any taxa in a given heatmap (red), the lower limit reflecting sequences above 0% abundance (green), and the midpoint limit (white) set to the true midpoint between 0% and the upper limit. In some instances, the midpoint limit was adjusted to highlight salient differences between the AUT-GI and Control-GI groups. Gray cells in all heatmaps reflect the complete absence of sequences detected for a given taxa in a given patient.

Operational Taxonomic Unit (OTU)-based analysis was carried out in MOTHUR (version 1.8.0) [95]. Filtered sequences generated from 454 pyrosequencing were aligned to the greengenes reference alignment (greengenes.lbl.gov), using the Needleman-Wunsch algorithm with the “align.seqs” function (ksize = 9). Pairwise genetic distances among the aligned sequences were calculated using the “dist.seqs” function (calc = onegap, countends = T). Sequences were assigned to OTUs (97% identity) using nearest neighbor clustering. Rarefaction curves to assess coverage and diversity (Shannon Diversity Index) were constructed in MOTHUR. For OTU analysis of Bacteroidete sequences, phylum level classification in RDP was used to subselect all Bacteroidete sequences, followed by OTU assignment at 97% identity. Representative sequences (defined as the sequence with the minimum distance to all other sequences in the OTU) from each OTU were obtained using the get.oturep command in MOTHUR. Representative sequences were classified using the nearest species match from Greengenes Blast (greengenes.lbl.gov) and NCBI BLAST alignment. OTU abundance by patient was expressed as percent relative abundance, determined by dividing the number of reads for an OTU in a given patient sample by the total number of bacterial reads obtained through pyrosequencing for that sample.

### Statistical analysis

Most of our data were not normally distributed, based on Kolmogorov-Smirnov test and evaluation of skewness and kurtosis; thus, the non-parametric Mann-Whitney U test was performed to evaluate differences between groups using StatView (Windows version 5.0.1; SAS Institute). The comparative results of gene expression and bacteria 16S rRNA gene levels were visualized as box-and-whisker plots showing: the median and the interquartile (midspread) range (boxes containing 50% of all values), the whiskers (representing the 25^th^ and 75^th^ percentiles), and the extreme data points (open circles). Chi-square test was used to evaluate between-group genotypes for adult-type hypolactasia, as well as differences in the frequency of atopic disease between groups. Kruskal-Wallis one-way analysis of variance was employed to assess significance of LCT mRNA expression levels split by genotype and group. To evaluate the effects of CDX2 and/or villin on enzyme and transporter levels and the effects of levels of enzymes and transporters on bacterial levels, multiple linear regression analyses were conducted. For details on multiple linear regression analyses see **Table 3**, **Table 4**, and **Text S2**. Significance was accepted for all analyses at *p*<0.05.

## Supporting Information

Figure S1 **Lactase genotyping.** (**A**) Distribution of genotypes for LCT-13910 and LCT-22018 polymorphisms between AUT-GI and Control-GI patients (chi-square test, *p* = 0.896). (**B**) Distribution of LCT mRNA expression in all individuals (AUT-GI and Control-GI) with the homozygous adult-type hypolactasia genotype (13910-C/C; 22018-G/G) compared to all individuals (AUT-GI and Control-GI) possessing at least one copy of the normal allele (heterozygous: 13910-C/T; 22018-G/A and homozygous: 13910-T/T; 22018-A/A); Mann-Whitney, *p* = 0.033. (**C**) Distribution of LCT mRNA expression levels split by genotype and group (AUT-GI and Control-GI); Kruskal-Wallis, *p* = 0.097. (**D**) Distribution of LCT mRNA expression for all patients possessing at least one copy of the normal (lactase persistence) allele for AUT-GI (n = 12) and Control-GI (n = 6); Mann-Whitney, p = 0.0246. Adult-type hypolactasia genotype is highlighted in red. *, *p*<0.05.
(TIF)

Figure S2 **Villin normalization and CDX2 expression stratified by total disaccharidase and transporter deficiencies.** Disaccharidase or transporter mRNA/villin mRNA ratios for SI (**A**; Mann-Whitney, *p* = 0.001), MGAM (**B**; Mann-Whitney, *p* = 0.001), LCT (**C**; Mann-Whitney, *p* = 0.005), SGLT1 (**D**; Mann-Whitney, *p* = 0.0008), and GLUT2 (**E**; Mann-Whitney, *p* = 0.002). (**F**) CDX2 mRNA expression in AUT-GI children stratified by number of total disaccharidase and transporter deficiencies [All 5 deficient (n = 10) or fewer than 5 deficient (n = 5)] compared to all Control-GI children (n = 7). AUT (All 5) vs. AUT (<5); Mann-Whitney, p = 0.037. AUT (All 5) vs. Control; Mann-Whitney, p = 0.064. *, p<0.05; **, *p*<0.01; ***, *p*<0.001; †, *p*<0.1 (trend).
(TIF)

Figure S3 **Diversity of AUT-GI and Control-GI phylotypes.** (**A–B**) Rarefaction curves assessing the completeness of sampling from pyrosequencing data obtained for individual AUT-GI (red) and Control-GI (blue) subjects' ileal (**A**) and cecal (**B**) biopsies. The y-axis indicates the number of OTUs detected (defined at 97% threshold for sequence similarity); the x-axis indicates the number of sequences sampled. (**C–D**) Rarefaction curves to estimate phylotype diversity, using the Shannon Diversity Index, from pyrosequencing data obtained for individual AUT-GI (red) and Control-GI (blue) subjects' ileal (**C**) and cecal (**D**) biopsies.
(TIF)

Figure S4 **OTU analysis of Bacteroidete phylotypes.** (**A–B**) Abundance distributions of the 12 most abundant Bacteroidete OTUs in ileal (**A**) and cecal (**B**) biopsies from AUT-GI and Control-GI children (bottom row displays cumulative levels of all 12 OTUs by patient). (**C–D**) Cumulative abundance of the 12 OTUs in ilea (**C**; Mann-Whitney, *p* = 0.008) and ceca (**D**; Mann-Whitney, *p* = 0.008) of AUT-GI and Control-GI children. (**E**) Classification of representative sequences obtained from each Bacteroidete OTU. Color code denotes the family-level, Ribosomal Database-derived taxonomic classification of each representative OTU sequence. **, p<0.01.
(TIF)

Figure S5 **Clostridiales/Bacteroidales ratios and abundance of Firmicutes assayed by pyrosequencing and real-time PCR.** (**A–B**) Order-level distribution of the Clostridiales/Bacteroidales ratio from pyrosequencing reads obtained from ileal (**A**; Mann-Whitney, *p* = 0.012) and cecal (**B**; Mann-Whitney, *p* = 0.032) biopsies from AUT-GI and Control-GI patients. (**C–D**) Phyla-level abundance of Firmicutes in the ilea (**C**; Mann-Whitney, *p* = 0.098) and ceca (**D**; Mann-Whitney, *p* = 0.148) of AUT-GI and Control-GI children obtained by pyrosequencing. (**E–F**) Phyla-level abundance of Firmicutes in the ilea (**E**; Mann-Whitney, *p* = 0.245) and ceca (**F**; Mann-Whitney, *p* = 0.053) of AUT-GI and Control-GI children obtained by real-time PCR. Copy number values for Firmicutes are normalized relative to total bacteria copy numbers. (**G–H**) Abundance of Clostridiales from ileal (**G**; Mann-Whitney, *p* = 0.072) and cecal (**H**; Mann-Whitney, *p* = 0.098) biopsies from AUT-GI and Control-GI patients obtained by pyrosequencing. *, *p*<0.05; †, *p*<0.1 (trend); n.s., not significant.
(TIF)

Figure S6 **Percent difference in abundance of Bacteroidetes, Firmicutes, and Proteobacteria in individual AUT-GI patients.** (**A–B**) Bar graphs indicating the percent difference in phylotype abundance for Bacteroidetes, Firmicutes, and Proteobacteria in AUT-GI patients (#1-15) relative to the Control-GI mean abundance for each of the three phylotypes obtained by pyrosequencing of ileal (**A**) and cecal (**B**) biopsies.
(TIF)

Figure S7 **Genus-level distribution of members of the families** ***Ruminococcaceae*** **and** ***Lachnospiraceae*** **.** (**A–B**) Heatmap representation of abundance distributions (by patient) of *Ruminococcaceae* and *Lachnospiraceae* genus members in ileal (**A**) and cecal (**B**) biopsies from AUT-GI and Control-GI patients. *, genus members contributing to the trend toward increased Firmicutes in AUT-GI children.
(TIF)

Figure S8 **Increased Betaproteobacteria in AUT-GI children is associated with total deficiencies in disaccharidases and hexose transporters and CDX2 mRNA expression.** (**A–B**) Abundance of Betaproteobacteria in AUT-GI children with deficiency in all 5 disaccharidases and transporters (All 5; n = 10), AUT-GI children with deficiency in fewer than 5 disaccharidases and transporters (<5; n = 5), and Control-GI children (n = 7) in ileum (**A**) and cecum (**B**). (**A**) Ileum: AUT-GI (All 5) vs. AUT-GI (<5), Mann-Whitney, p = 0.028; AUT-GI (All 5) vs. Control-GI, Mann-Whitney, p = 0.015; AUT-GI (<5) vs. Control-GI, Mann-Whitney, p = 0.935. (**B**) Cecum: AUT-GI (All 5) vs. AUT-GI (<5), Mann-Whitney, p = 0.014; AUT-GI (All 5) vs. Control-GI, Mann-Whitney, p = 0.006; AUT-GI (<5) vs. Control-GI, Mann-Whitney, p = 0.808. (**C–D**) Ileal CDX2 mRNA expression in AUT-GI children with Betaproteobacteria above the 75^th^ percentile of Control-GI children [AUT (+) β-proteo.], AUT-GI children with Betaproteobacteria levels below the 75^th^ percentile of Control-GI children [AUT (−) β-proteo.], and Control-GI children in ileum (**C**) and cecum (**D**). (**C**) Ileum: AUT (+) β-proteo. (n = 8) vs. AUT (−) β-proteo. (n = 7), Mann-Whitney, p = 0.037; AUT (+) β-proteo. vs. Control-GI (n = 7), Mann-Whitney, p = 0.064; AUT (−) β-proteo. vs. Control-GI, Mann-Whitney, p = 0.749. (**D**) Cecum: AUT (+) β-proteo. (n = 10) vs. AUT (−) β-proteo. (n = 5), Mann-Whitney, p = 0.028; AUT (+) β-proteo. vs. Control-GI (n = 7), Mann-Whitney, p = 0.097; AUT (−) β-proteo. vs. Control-GI, Mann-Whitney, p = 0.808. *, p<0.05; **, *p*<0.01; †, *p*<0.1 (trend); n.s., not significant.
(TIF)

Table S1 **Reported comorbid conditions, food allergies, and medication use by patient.**
(DOC)

Table S2 **Reported behavioral regression in AUT-GI children.** Legend: *ADI-R*, Autism Diagnostic Interview-Revised; *CDI*, MacArthur Communicative Development Inventory; *CPEA*, Collaborative Program for Excellence in Autism.
(TIF)

Table S3 **Fold-change in gene expression in AUT-GI children.** Legend: Fold-change values were calculated relative to the mean expression level obtained for all Control-GI children for each gene. Expression levels for individual patients that were at least two-fold increased (>2) or decreased (<0.5) relative to the Control-GI mean are highlighted in red and green, respectively.
(TIF)

Table S4 **Percent change in bacterial levels in AUT-GI children.** Legend: Percent change values were calculated for real-time PCR and ratio data relative to the mean levels obtained for all Control-GI children for each bacterial variable. Percent difference values were calculated for pyrosequencing data by subtracting the mean percent abundance of Control-GI children from the percent abundance of each AUT-GI patient for each variable.
(TIF)

Table S5 **Evaluation of confounding effects attributed to the use of (A) probiotics, (B) proton-pump inhibitors, and (C) antibiotics.**
(DOC)

Table S6 **Real-time PCR primers and probes used for gene expression and bacterial quantitative analysis.**
(TIF)

Text S1 **Supporting Results.**
(DOC)

Text S2 **Supporting Methods.**
(DOC)
