# Supplementary figures and images for: Impaired Carbohydrate Digestion and Transport and Mucosal Dysbiosis in the Intestines of Children with Autism and Gastrointestinal Disturbances
Source: PLoS One. 2011 Sep 16;6(9):e24585. doi: 10.1371/journal.pone.0024585 (PMC3174969; doi:10.1371/journal.pone.0024585)

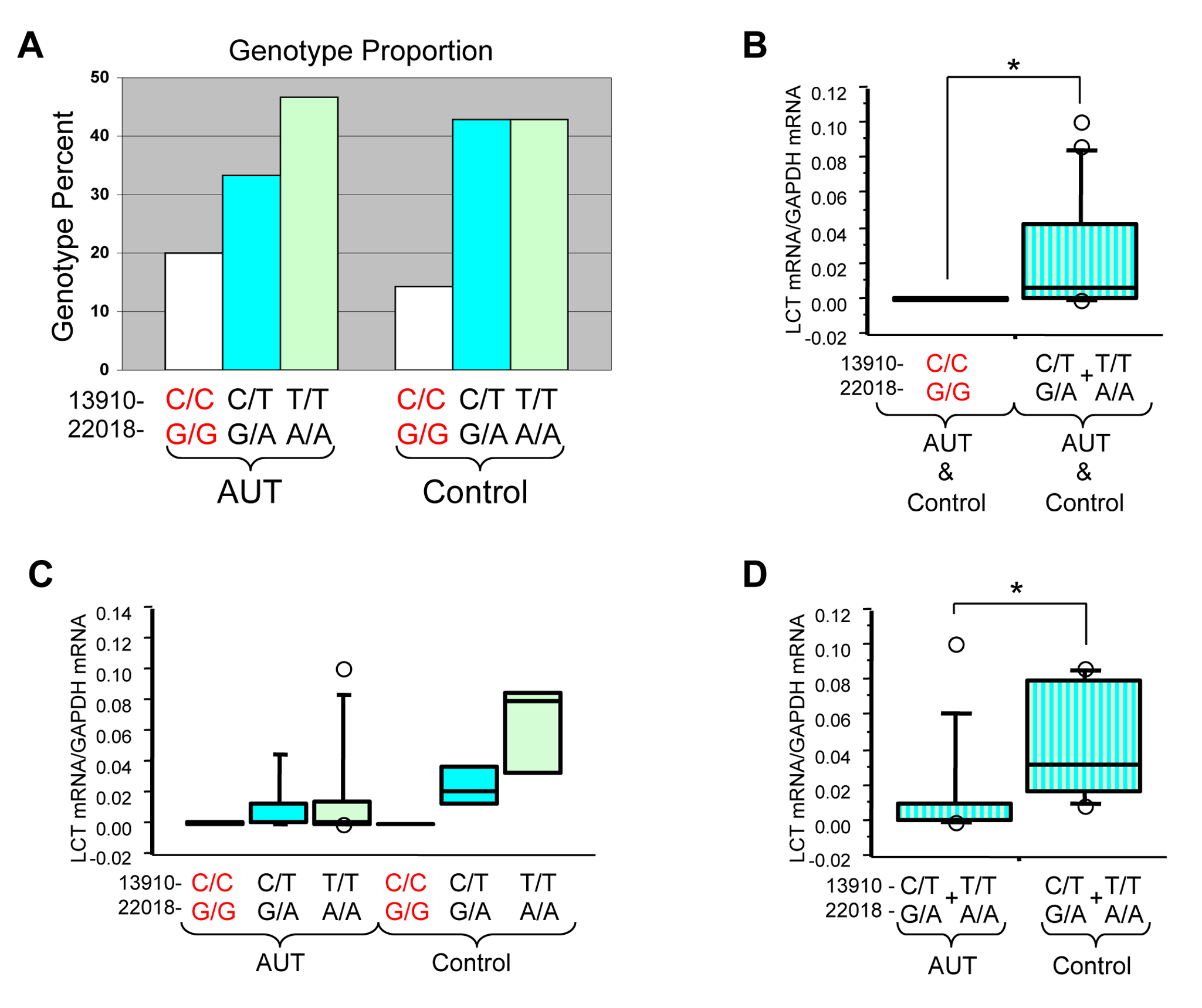

Supplement: Figure S1 — Lactase genotyping. (A) Distribution of genotypes for LCT-13910 and LCT-22018 polymorphisms between AUT-GI and Control-GI patients (chi-square test, p = 0.896). (B) Distribution of LCT mRNA expression in all individuals (AUT-GI and Control-GI) with the homozygous adult-type hypolactasia genotype (13910-C/C; 22018-G/G) compared to all individuals (AUT-GI and Control-GI) possessing at least one copy of the normal allele (heterozygous: 13910-C/T; 22018-G/A and homozygous: 13910-T/T; 22018-A/A); Mann-Whitney, p = 0.033. (C) Distribution of LCT mRNA expression levels split by genotype and group (AUT-GI and Control-GI); Kruskal-Wallis, p = 0.097. (D) Distribution of LCT mRNA expression for all patients possessing at least one copy of the normal (lactase persistence) allele for AUT-GI (n = 12) and Control-GI (n = 6); Mann-Whitney, p = 0.0246. Adult-type hypolactasia genotype is highlighted in red. *, p<0.05. (TIF) [file pone.0024585.s001.tif]

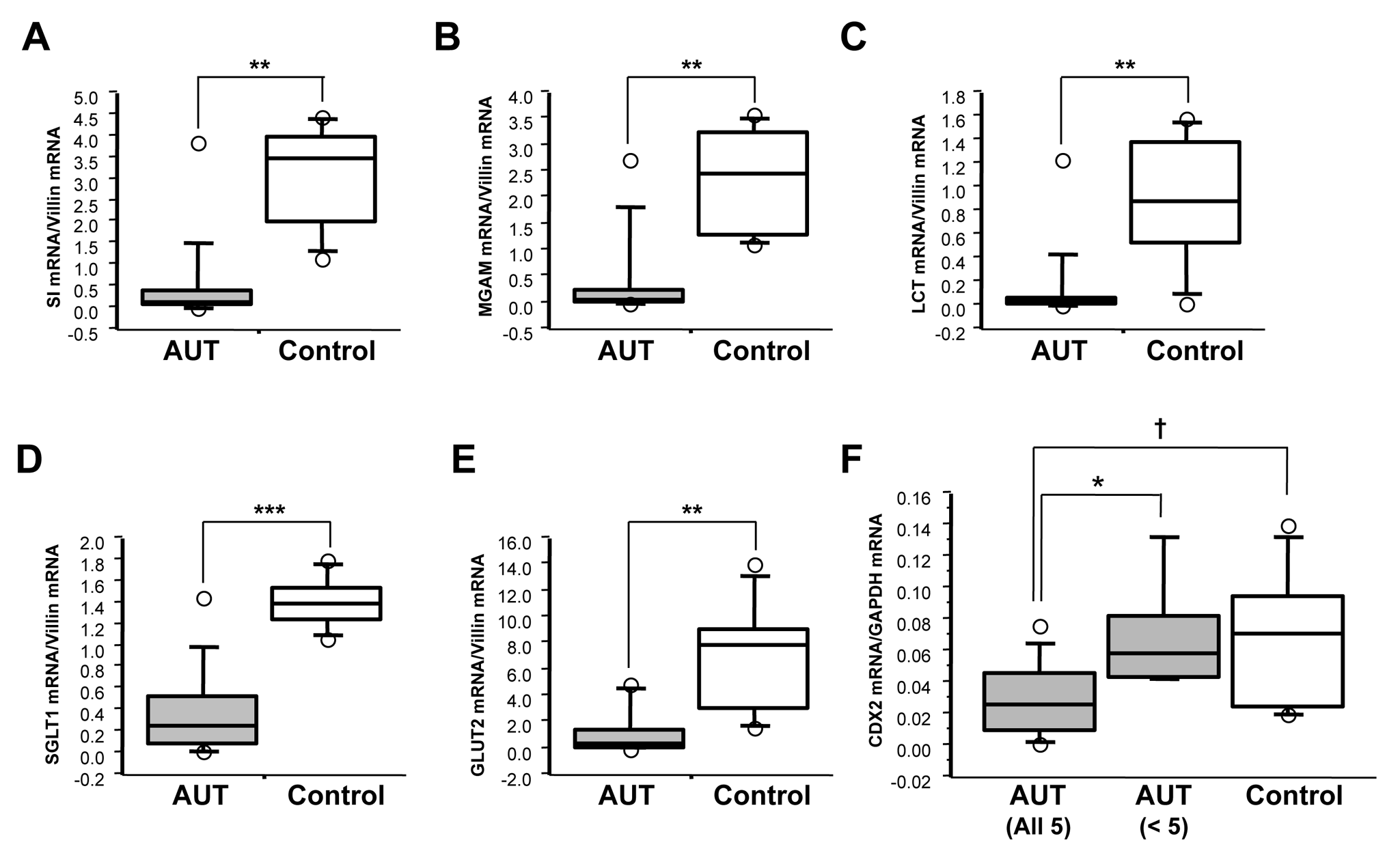

Supplement: Figure S2 — Villin normalization and CDX2 expression stratified by total disaccharidase and transporter deficiencies. Disaccharidase or transporter mRNA/villin mRNA ratios for SI (A; Mann-Whitney, p = 0.001), MGAM (B; Mann-Whitney, p = 0.001), LCT (C; Mann-Whitney, p = 0.005), SGLT1 (D; Mann-Whitney, p = 0.0008), and GLUT2 (E; Mann-Whitney, p = 0.002). (F) CDX2 mRNA expression in AUT-GI children stratified by number of total disaccharidase and transporter deficiencies [All 5 deficient (n = 10) or fewer than 5 deficient (n = 5)] compared to all Control-GI children (n = 7). AUT (All 5) vs. AUT (<5); Mann-Whitney, p = 0.037. AUT (All 5) vs. Control; Mann-Whitney, p = 0.064. *, p<0.05; **, p<0.01; ***, p<0.001; †, p<0.1 (trend). (TIF) [file pone.0024585.s002.tif]

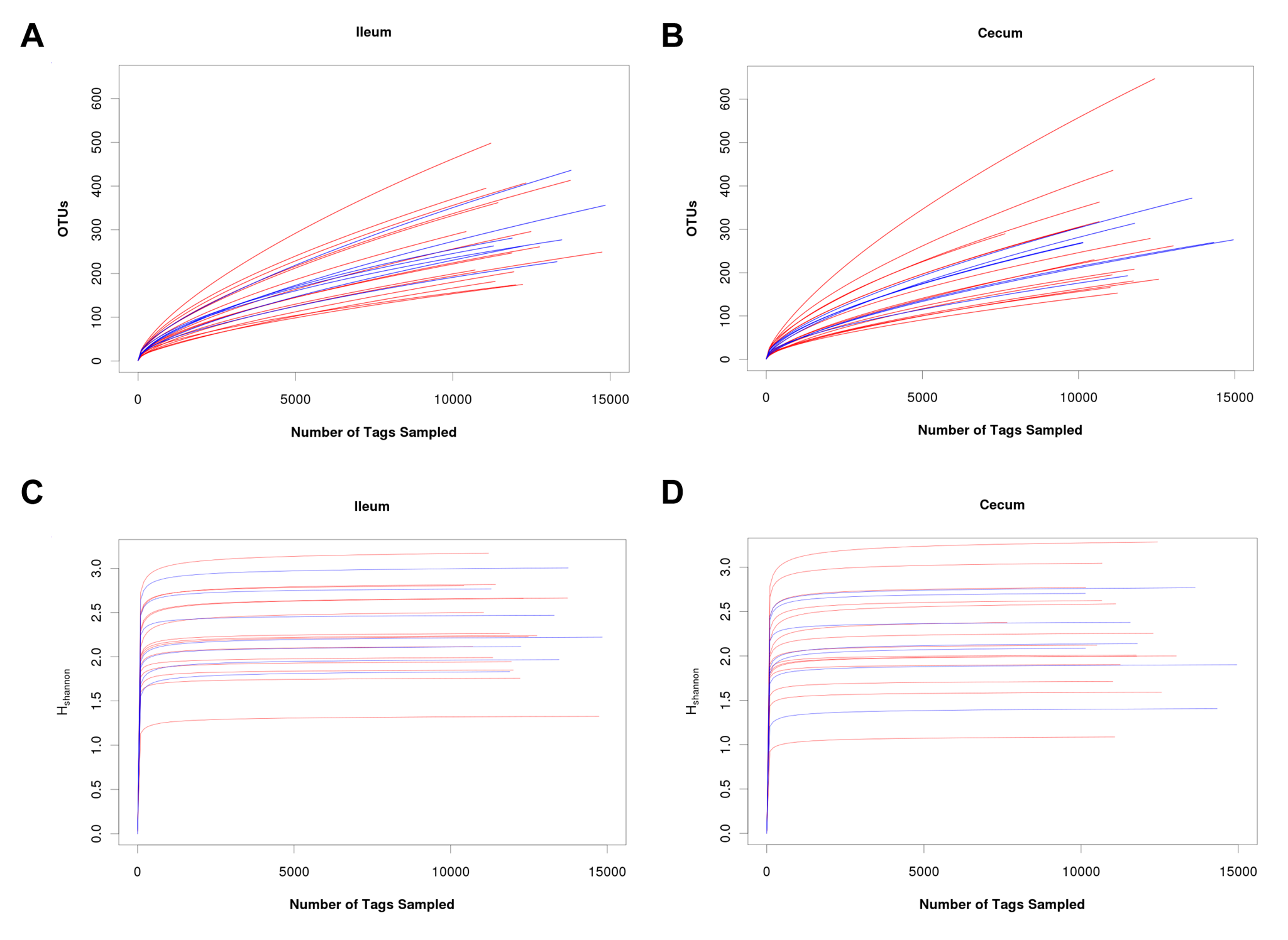

Supplement: Figure S3 — Diversity of AUT-GI and Control-GI phylotypes. (A–B) Rarefaction curves assessing the completeness of sampling from pyrosequencing data obtained for individual AUT-GI (red) and Control-GI (blue) subjects' ileal (A) and cecal (B) biopsies. The y-axis indicates the number of OTUs detected (defined at 97% threshold for sequence similarity); the x-axis indicates the number of sequences sampled. (C–D) Rarefaction curves to estimate phylotype diversity, using the Shannon Diversity Index, from pyrosequencing data obtained for individual AUT-GI (red) and Control-GI (blue) subjects' ileal (C) and cecal (D) biopsies. (TIF) [file pone.0024585.s003.tif]

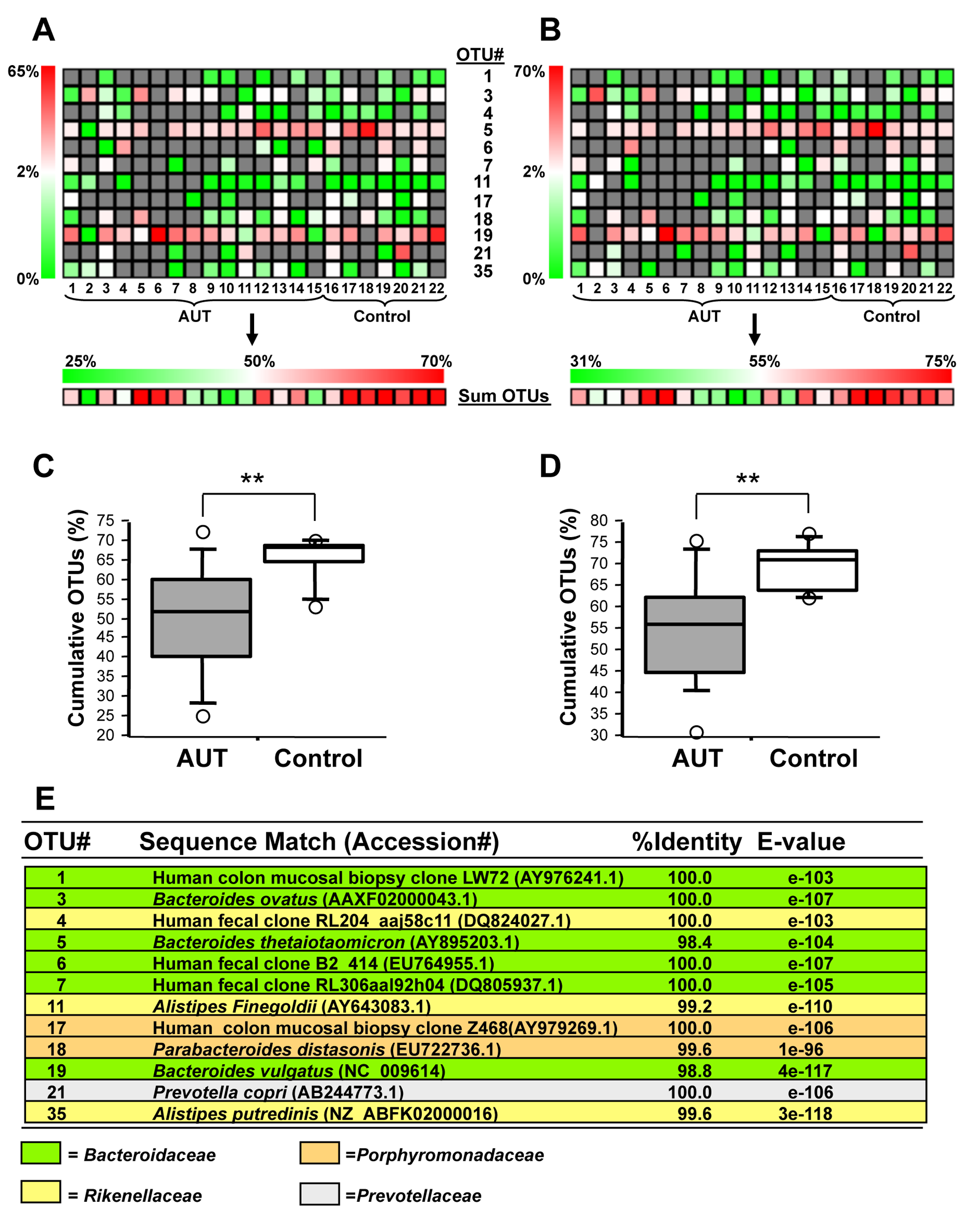

Supplement: Figure S4 — OTU analysis of Bacteroidete phylotypes. (A–B) Abundance distributions of the 12 most abundant Bacteroidete OTUs in ileal (A) and cecal (B) biopsies from AUT-GI and Control-GI children (bottom row displays cumulative levels of all 12 OTUs by patient). (C–D) Cumulative abundance of the 12 OTUs in ilea (C; Mann-Whitney, p = 0.008) and ceca (D; Mann-Whitney, p = 0.008) of AUT-GI and Control-GI children. (E) Classification of representative sequences obtained from each Bacteroidete OTU. Color code denotes the family-level, Ribosomal Database-derived taxonomic classification of each representative OTU sequence. **, p<0.01. (TIF) [file pone.0024585.s004.tif]

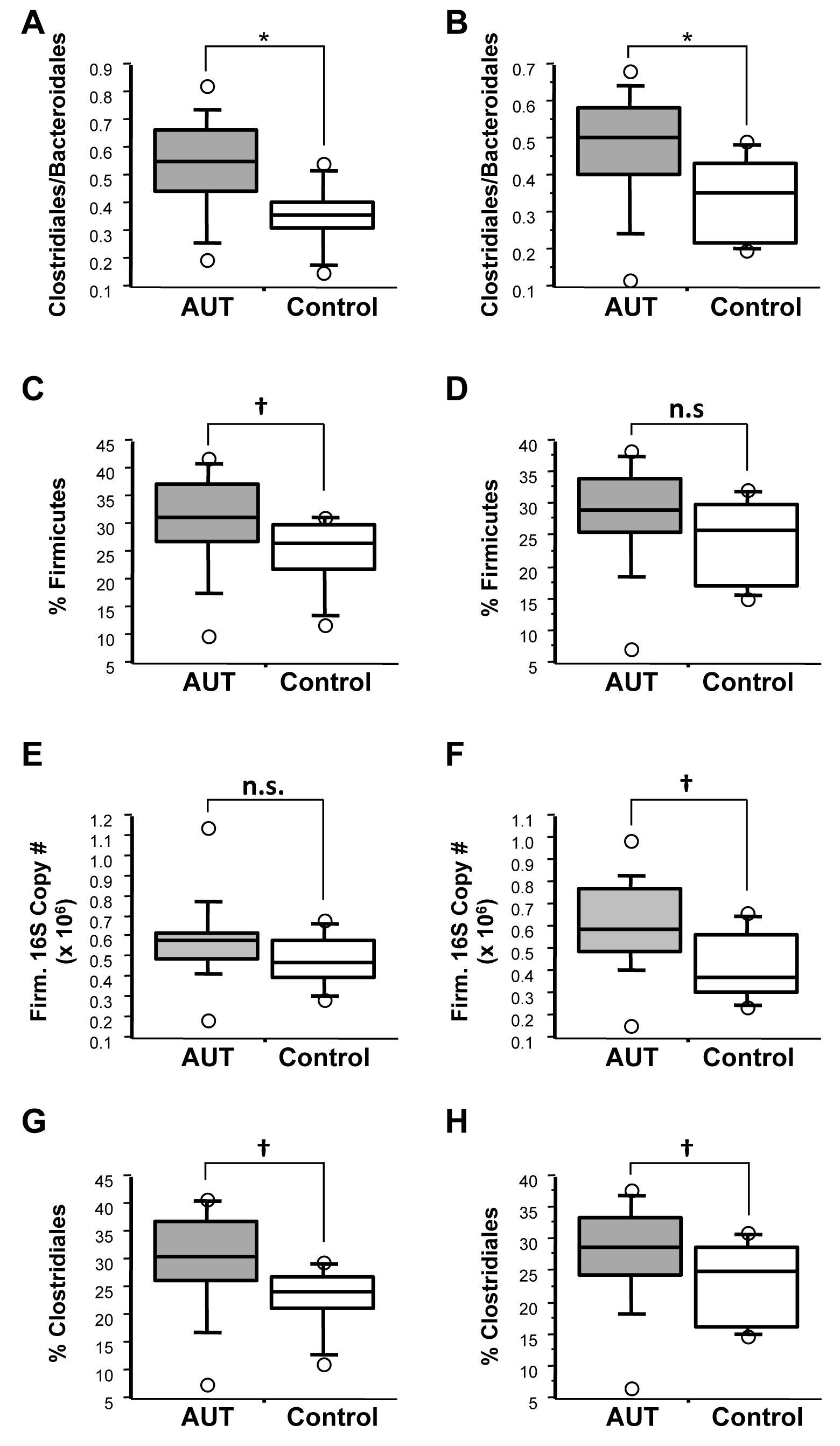

Supplement: Figure S5 — Clostridiales/Bacteroidales ratios and abundance of Firmicutes assayed by pyrosequencing and real-time PCR. (A–B) Order-level distribution of the Clostridiales/Bacteroidales ratio from pyrosequencing reads obtained from ileal (A; Mann-Whitney, p = 0.012) and cecal (B; Mann-Whitney, p = 0.032) biopsies from AUT-GI and Control-GI patients. (C–D) Phyla-level abundance of Firmicutes in the ilea (C; Mann-Whitney, p = 0.098) and ceca (D; Mann-Whitney, p = 0.148) of AUT-GI and Control-GI children obtained by pyrosequencing. (E–F) Phyla-level abundance of Firmicutes in the ilea (E; Mann-Whitney, p = 0.245) and ceca (F; Mann-Whitney, p = 0.053) of AUT-GI and Control-GI children obtained by real-time PCR. Copy number values for Firmicutes are normalized relative to total bacteria copy numbers. (G–H) Abundance of Clostridiales from ileal (G; Mann-Whitney, p = 0.072) and cecal (H; Mann-Whitney, p = 0.098) biopsies from AUT-GI and Control-GI patients obtained by pyrosequencing. *, p<0.05; †, p<0.1 (trend); n.s., not significant. (TIF) [file pone.0024585.s005.tif]

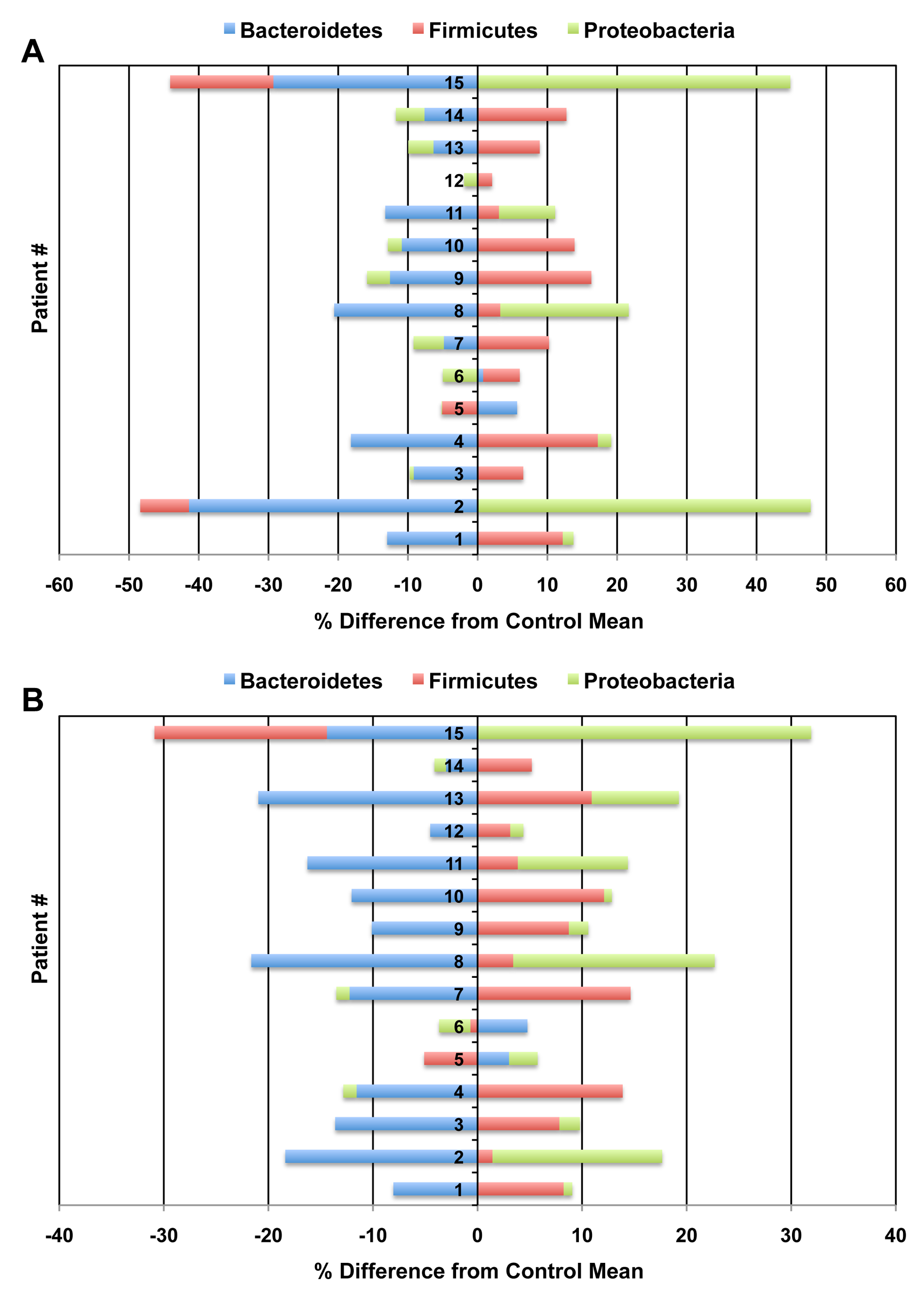

Supplement: Figure S6 — Percent difference in abundance of Bacteroidetes, Firmicutes, and Proteobacteria in individual AUT-GI patients. (A–B) Bar graphs indicating the percent difference in phylotype abundance for Bacteroidetes, Firmicutes, and Proteobacteria in AUT-GI patients (#1-15) relative to the Control-GI mean abundance for each of the three phylotypes obtained by pyrosequencing of ileal (A) and cecal (B) biopsies. (TIF) [file pone.0024585.s006.tif]

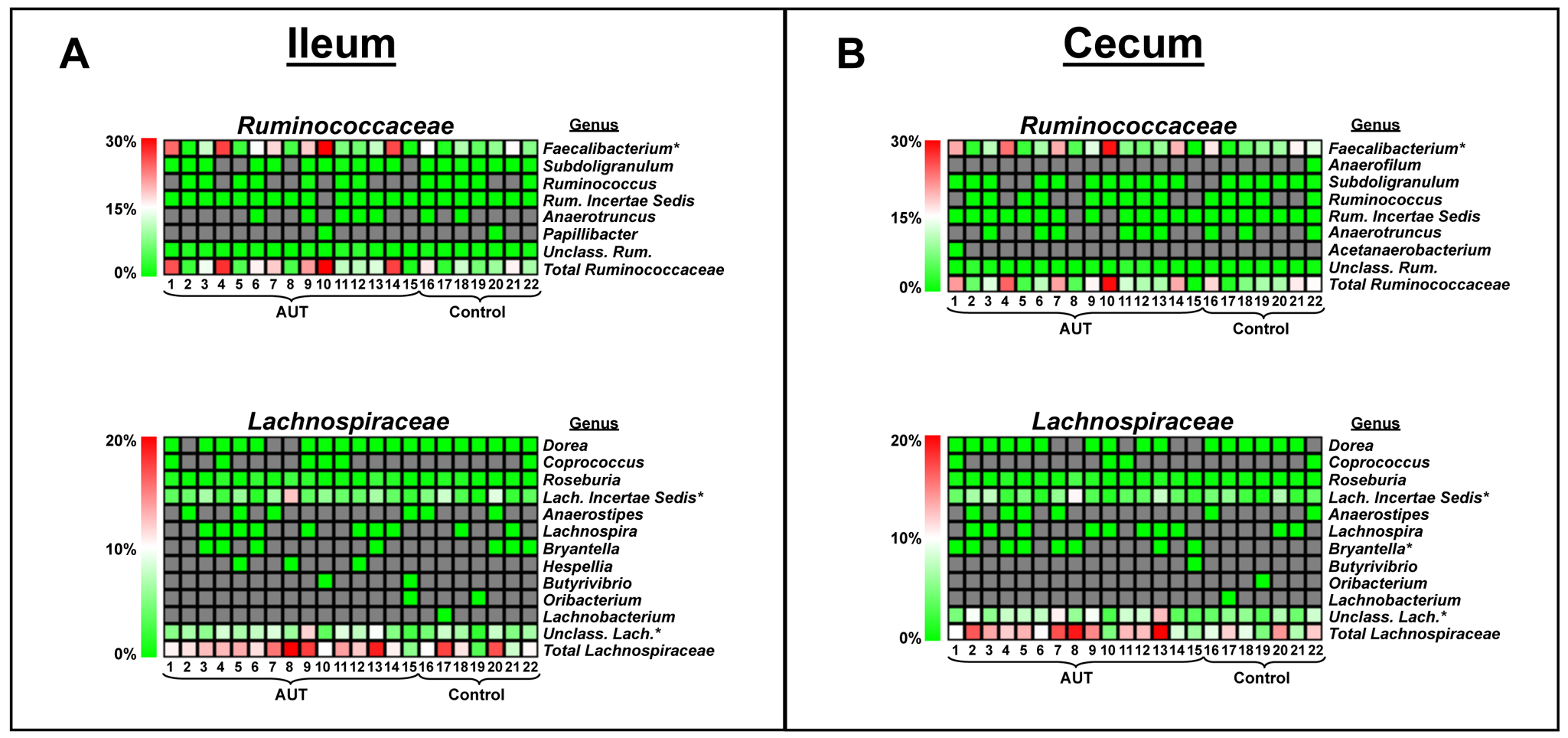

Supplement: Figure S7 — Genus-level distribution of members of the families Ruminococcaceae and Lachnospiraceae . (A–B) Heatmap representation of abundance distributions (by patient) of Ruminococcaceae and Lachnospiraceae genus members in ileal (A) and cecal (B) biopsies from AUT-GI and Control-GI patients. *, genus members contributing to the trend toward increased Firmicutes in AUT-GI children. (TIF) [file pone.0024585.s007.tif]

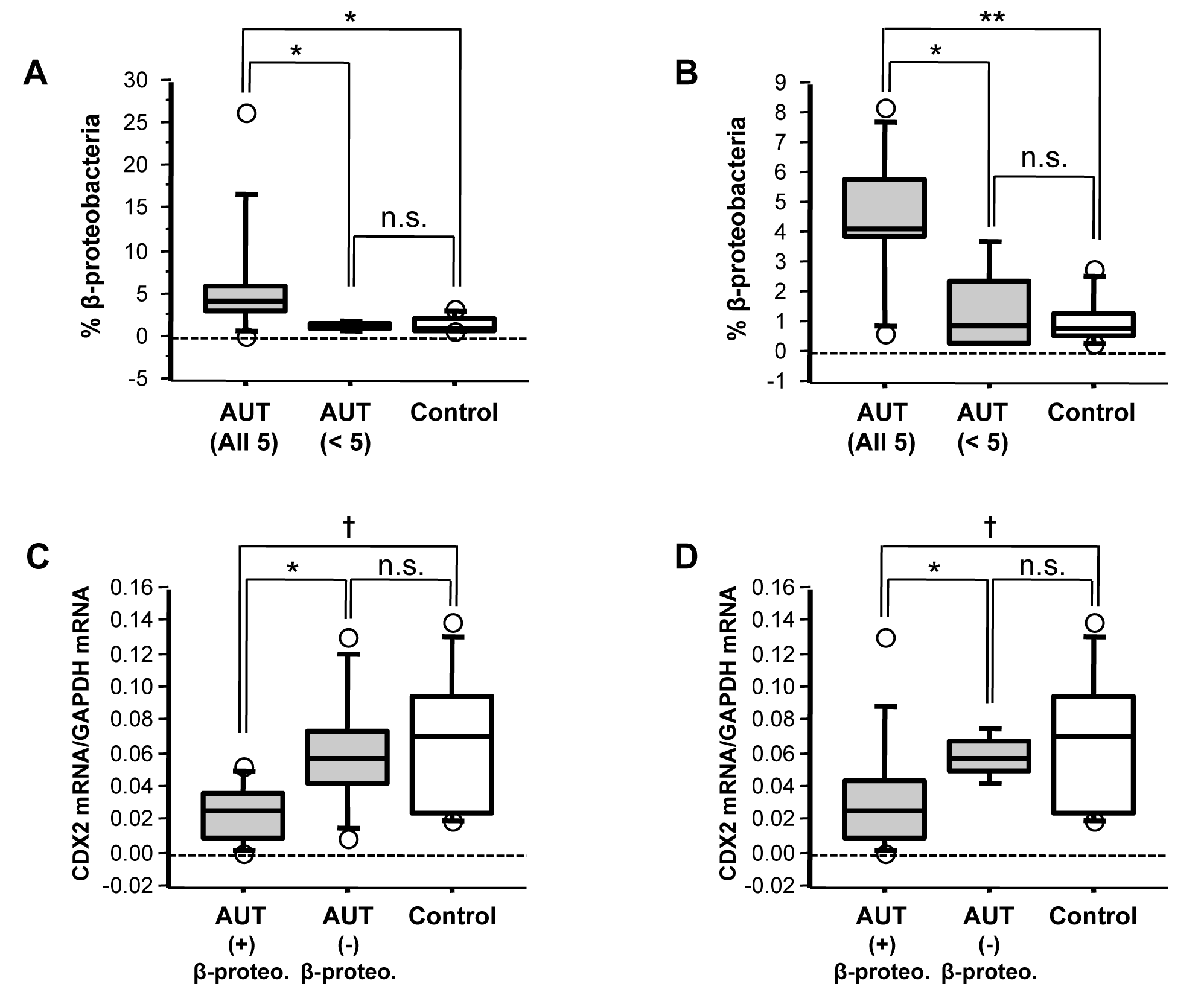

Supplement: Figure S8 — Increased Betaproteobacteria in AUT-GI children is associated with total deficiencies in disaccharidases and hexose transporters and CDX2 mRNA expression. (A–B) Abundance of Betaproteobacteria in AUT-GI children with deficiency in all 5 disaccharidases and transporters (All 5; n = 10), AUT-GI children with deficiency in fewer than 5 disaccharidases and transporters (<5; n = 5), and Control-GI children (n = 7) in ileum (A) and cecum (B). (A) Ileum: AUT-GI (All 5) vs. AUT-GI (<5), Mann-Whitney, p = 0.028; AUT-GI (All 5) vs. Control-GI, Mann-Whitney, p = 0.015; AUT-GI (<5) vs. Control-GI, Mann-Whitney, p = 0.935. (B) Cecum: AUT-GI (All 5) vs. AUT-GI (<5), Mann-Whitney, p = 0.014; AUT-GI (All 5) vs. Control-GI, Mann-Whitney, p = 0.006; AUT-GI (<5) vs. Control-GI, Mann-Whitney, p = 0.808. (C–D) Ileal CDX2 mRNA expression in AUT-GI children with Betaproteobacteria above the 75th percentile of Control-GI children [AUT (+) β-proteo.], AUT-GI children with Betaproteobacteria levels below the 75th percentile of Control-GI children [AUT (−) β-proteo.], and Control-GI children in ileum (C) and cecum (D). (C) Ileum: AUT (+) β-proteo. (n = 8) vs. AUT (−) β-proteo. (n = 7), Mann-Whitney, p = 0.037; AUT (+) β-proteo. vs. Control-GI (n = 7), Mann-Whitney, p = 0.064; AUT (−) β-proteo. vs. Control-GI, Mann-Whitney, p = 0.749. (D) Cecum: AUT (+) β-proteo. (n = 10) vs. AUT (−) β-proteo. (n = 5), Mann-Whitney, p = 0.028; AUT (+) β-proteo. vs. Control-GI (n = 7), Mann-Whitney, p = 0.097; AUT (−) β-proteo. vs. Control-GI, Mann-Whitney, p = 0.808. *, p<0.05; **, p<0.01; †, p<0.1 (trend); n.s., not significant. (TIF) [file pone.0024585.s008.tif]

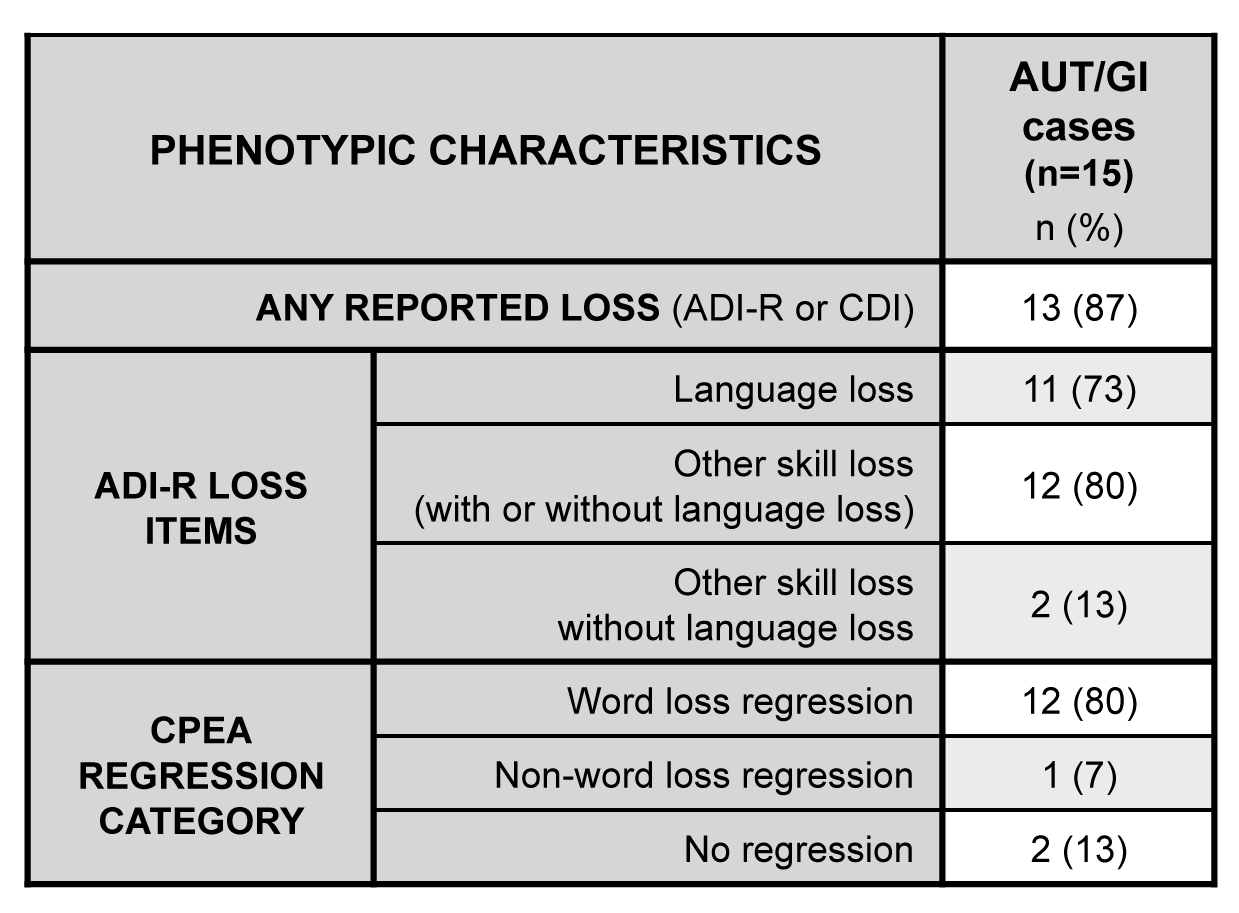

Supplement: Table S2 — Reported behavioral regression in AUT-GI children. Legend: ADI-R, Autism Diagnostic Interview-Revised; CDI, MacArthur Communicative Development Inventory; CPEA, Collaborative Program for Excellence in Autism. (TIF) [file pone.0024585.s010.tif]

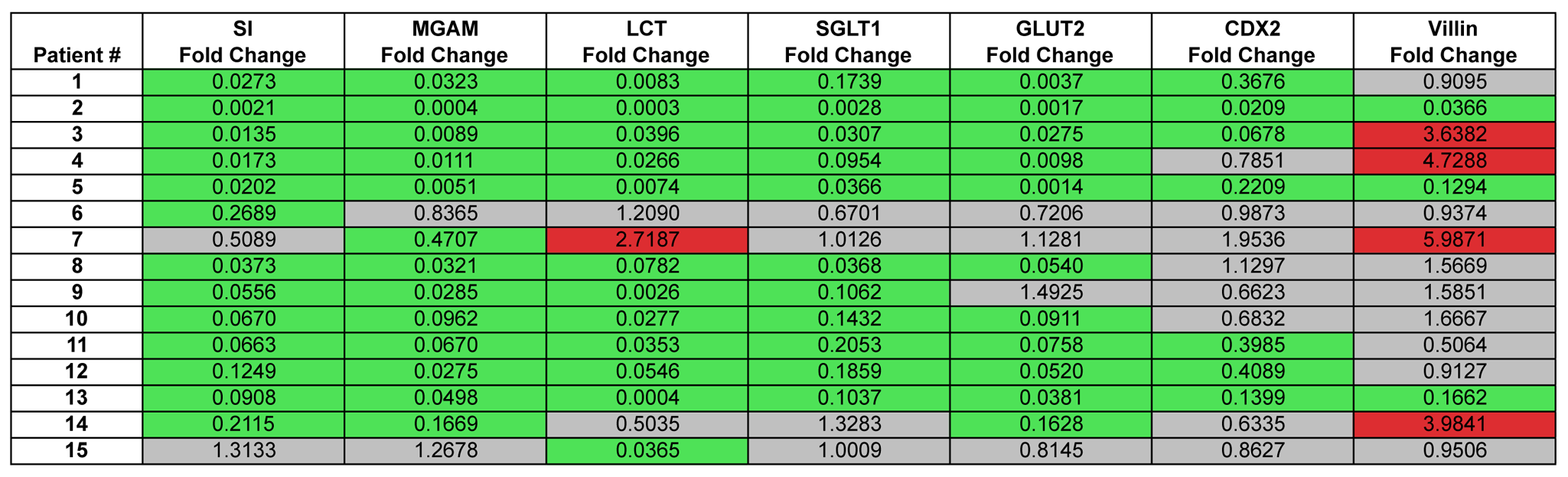

Supplement: Table S3 — Fold-change in gene expression in AUT-GI children. Legend: Fold-change values were calculated relative to the mean expression level obtained for all Control-GI children for each gene. Expression levels for individual patients that were at least two-fold increased (>2) or decreased (<0.5) relative to the Control-GI mean are highlighted in red and green, respectively. (TIF) [file pone.0024585.s011.tif]

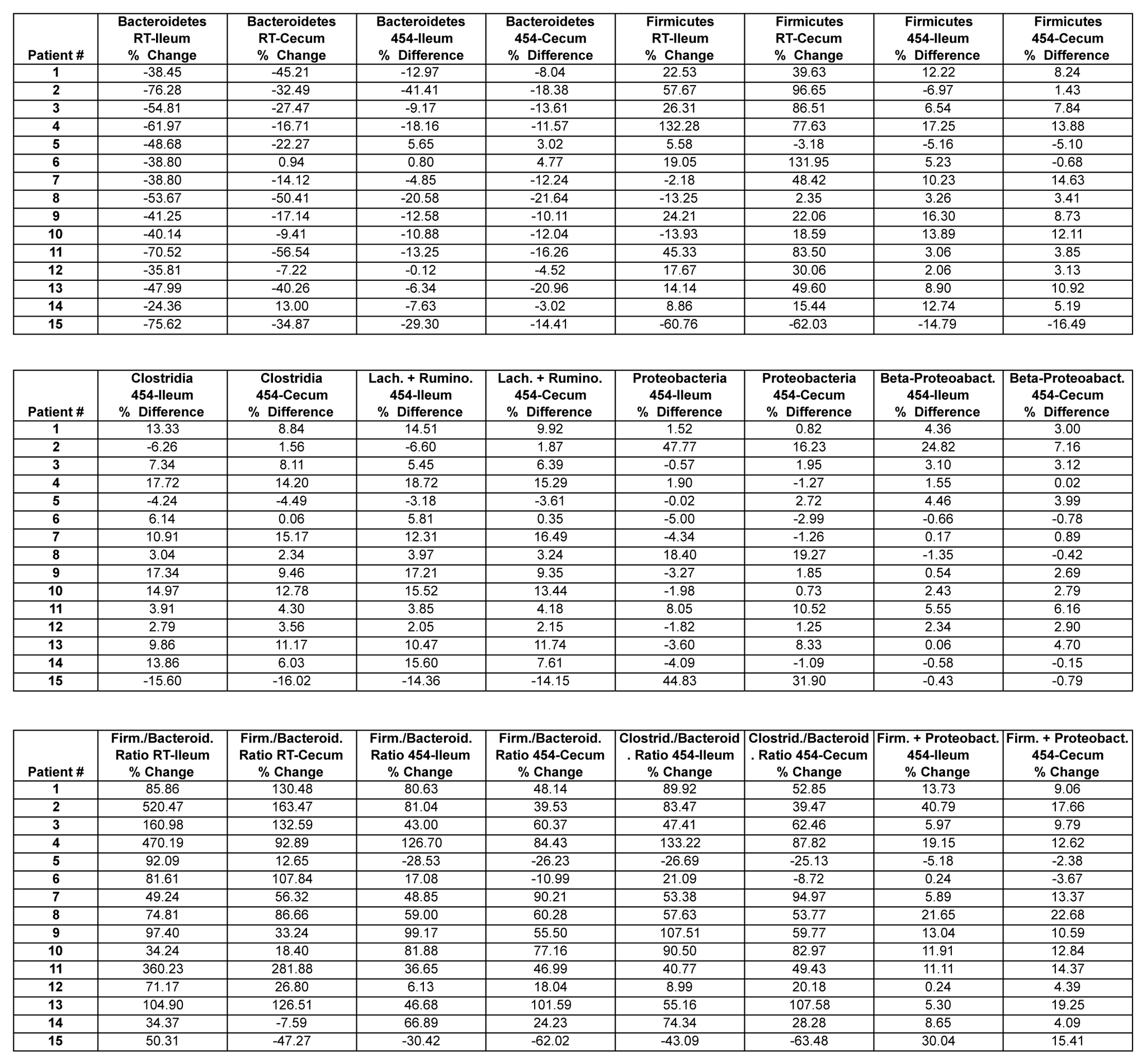

Supplement: Table S4 — Percent change in bacterial levels in AUT-GI children. Legend: Percent change values were calculated for real-time PCR and ratio data relative to the mean levels obtained for all Control-GI children for each bacterial variable. Percent difference values were calculated for pyrosequencing data by subtracting the mean percent abundance of Control-GI children from the percent abundance of each AUT-GI patient for each variable. (TIF) [file pone.0024585.s012.tif]

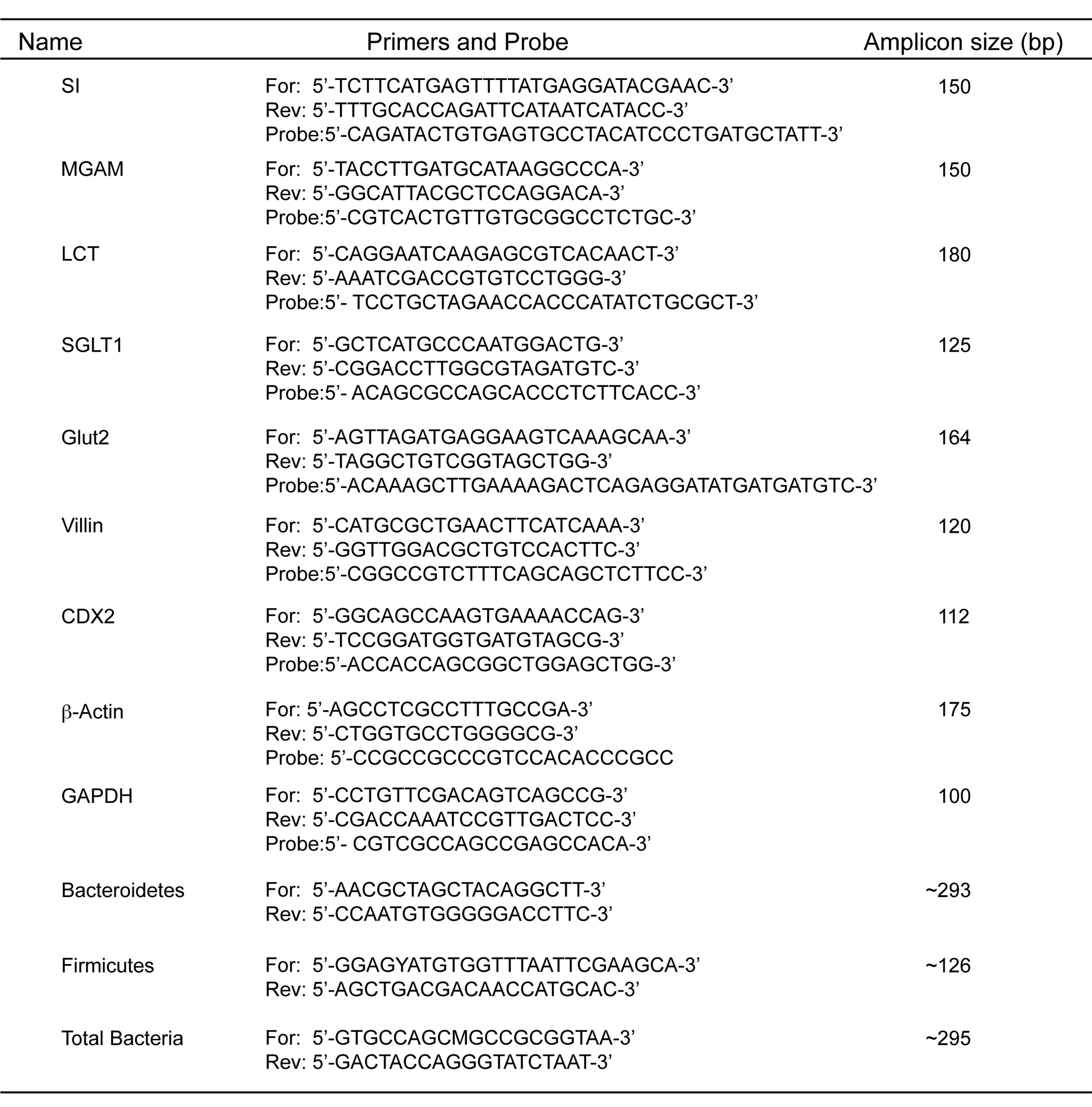

Supplement: Table S6 — Real-time PCR primers and probes used for gene expression and bacterial quantitative analysis. (TIF) [file pone.0024585.s014.tif]
