# Supplementary material for: Impaired Carbohydrate Digestion and Transport and Mucosal Dysbiosis in the Intestines of Children with Autism and Gastrointestinal Disturbances
Source: PLoS One. 2011 Sep 16;6(9):e24585. doi: 10.1371/journal.pone.0024585 (PMC3174969; doi:10.1371/journal.pone.0024585)
Supplement: Table S1 — Reported comorbid conditions, food allergies, and medication use by patient. (DOC) [file pone.0024585.s009.doc]

| **Patient ID** | **Group** | **Current Comorbid Conditions** | **Food Allergy Reported** | **Medications** |
| --- | --- | --- | --- | --- |
| 1 | AUT-GI | asthma, atopic dermatitis, celiac disease, movement disorder, myopathy | milk, gluten, eggs, peanuts, tree nuts, soy, corn, peas | Vitamin B1, B2, B3, B6, B9, B12, C, E; Ca, Zn, Fish oil, Omega-3-fatty acids, Probiotic, Ibuprofen, Lansoprazole, Montelukast sodium, Levalbuterol inhaler, Albuterol inhaler |
| 2 | AUT-GI | allergic rhinitis | milk, gluten, eggs | Vitamin C; MVM, Ca/Mg supplement, Omeprazole |
| 3 | AUT-GI | IBD | milk, gluten, dyes | Vitamin B12, C; MVM, Ca/Mg supplement, Zn, flaxseed oil, antifungal herbal agent, digestive enzymes |
| 4 | AUT-GI | allergic rhinitis, asthma, atopic dermatitis, migraine | casein, gluten | Vitamin A, C, Methyl-B12, Folinic acid; MVM, Ca/Mg supplement, Zn, Mb, Fish oil, Omega-3-fatty acids, SAMe, Inositol, Selenomethionine, Trimethylglycine, 5-methyl-tetrahydrofolate, Transdermal glutathione, MgSO4 cream, Zn soy cream, DMAE, DMPS, Alpha lipoic acid, Montelukast sodium |
| 5 | AUT-GI | atopic dermatitis | lactose | MVM |
| 6 | AUT-GI | allergic rhinitis, frequent URI, epilepsy | gluten, corn, soy | Vitamin D; Ca, Zn, Mg, P, Flaxseed oil, Probiotic, Artichoke extract, Sarsaparilla extract, Wasabi powder, Lipase, Amylase, Protease |
| 7 | AUT-GI | allergic rhinitis, frequent otitis media | milk, gluten, sweet potatoes, oranges, berries | Folinic acid; MVM, Ca/Mg supplement, Trimethylglycine, Lipase, Amylase, Protease, Cellulase, Lactase |
| 8 | AUT-GI | none | none reported | Vitamin B complex, L-carnitine, Lipase, Amylase, Protease, Diphenhydramine, Acetaminophen, Ibuprofen, Melatonin, Sertraline, Valproic acid |
| 9 | AUT-GI | none | none reported | MVM, Ca |
| 10 | AUT-GI | none | none reported | Omeprazole |
| 11 | AUT-GI | atopic dermatitis | cow's milk, goat's milk, barley, carrots, bananas, cantelope, coffee, cranberry, lamb, lettuce | Flaxseed oil, Coenzyme Q10, Cell signal enhancers (CSE-14, 15), Probiotic, Lipase |
| 12 | AUT-GI | Epstein-Barr virus infection | dairy, wheat, salicylates, phenols | Methyl-B12, DMSA, Amphoterecin B |
| 13 | AUT-GI | asthma | dairy, wheat, yeast | Vitamin B12; Ca/Mg supplement, Zn, Probiotic, Clonidine, Secretin |
| 14 | AUT-GI | none | none reported | MVM, F |
| 15 | AUT-GI | none | none reported | Lipase, Amylase, Protease, Milk of magnesia, Lansoprazole |
| 16 | Control-GI | allergic rhinitis, asthma, atopic dermatitis, frequent sinusitis | none reported | MVM, Montelukast sodium, Fluticasone propionate, Lansoprazole, Amoxicillin |
| 17 | Control-GI | atopic dermatitis | none reported | Ca citrate, Mg/amino acid complex, Hydroxyzine, Budesonide, Prednisolone, Montelukast sodium, Levalbuterol inhaler, Tacrolimus |
| 18 | Control-GI | asthma | dairy, peanuts | Ibuprofen |
| 19 | Control-GI | asthma, atopic dermatitis, IBD, dysphagia, microcytic anemia, pancreatic insufficiency | milk, wheat, eggs, oats, salmon, soy, peanut, tree nut, chicken, turkey, beef, broccoli, cabbage, lentils, legumes | Lipase, Amylase, Protease, Diphenhydramine, Cetirizine hydrochloride, Omeprazole, Budesonide, Montelukast sodium, Levalbuterol inhaler |
| 20 | Control-GI | allergic rhinitis, asthma, atopic dermatitis | dairy, gluten, eggs, soy, citrus | Vitamin B12, Fish oil, Milk thistle, DMSA, Allithiamine |
| 21 | Control-GI | asthma | dairy, wheat, eggs, fruit | Probiotic |
| 22 | Control-GI | allergic rhinitis, vitiligo | dairy, wheat, eggs, peanuts, beef | none reported |

IBD- Inflammatory Bowel Disease; URI- Upper respiratory tract infection; MVM-multivitamin with minerals; SAMe- S-adenosylmethionine; DMAE- dimethylaminoethanol; DMPS- 2,3-Dimercapto-1-propanesulfonic acid; DMSA- Dimercaptosuccinic acid
