# Supplementary material for: Impaired Carbohydrate Digestion and Transport and Mucosal Dysbiosis in the Intestines of Children with Autism and Gastrointestinal Disturbances
Source: PLoS One. 2011 Sep 16;6(9):e24585. doi: 10.1371/journal.pone.0024585 (PMC3174969; doi:10.1371/journal.pone.0024585)
Supplement: Table S5 — Evaluation of confounding effects attributed to the use of (A) probiotics, (B) proton-pump inhibitors, and (C) antibiotics. (DOC) [file pone.0024585.s013.doc]

A.

|  | **Probiotics (Pb)** | |
| --- | --- | --- |
|  | **AUT(-Pb) vs. Control(-Pb)^a^, p-value^MW^, [effect in AUT(-Pb)]** | **AUT(-Pb) vs. AUT(+Pb)^b^, p-value^MW^, [effect in AUT(+Pb)]** |
| **SI** | 0.007**, [decreased] | 0.602, [no change] |
| **MGAM** | 0.007**, [decreased] | 0.240, [no change] |
| **LCT** | 0.012*, [decreased] | 0.695, [no change] |
| **SGLT1** | 0.021*, [decreased] | 0.433, [no change] |
| **GLUT2** | 0.021*, [decreased] | 0.794, [no change] |
| **Bacteroidetes IL(RT)** | 0.009**, [decreased] | 0.602, [no change] |
| **Bacteroidetes CEC(RT)** | 0.056†, [decreased] | 0.192, [no change] |
| **Bacteroidetes IL(454)** | 0.035*, [decreased] | 0.602, [no change] |
| **Bacteroidetes CEC(454)** | 0.009**, [decreased] | 0.999, [no change] |
| **Firm./Bacteroid. Ratio IL(RT)** | 0.004**, [increased] | 0.361, [no change] |
| **Firm. /Bacteroid. Ratio CEC(RT)** | 0.159, [no change] | 0.037*, [increased] |
| **Firm./Bacteroid. Ratio IL(454)** | 0.070†, [increased] | 0.514, [no change] |
| **Firm./Bacteroid. Ratio CEC(454)** | 0.056†, [increased] | 0.896, [no change] |
| **Clostridiales/Bacteroidales IL(454)** | 0.044*, [increased] | 0.695, [no change] |
| **Clostridiales/Bacteroidales CEC(454)** | 0.070†, [increased] | 0.896, [no change] |
| **Beta-proteobacteria CEC(454)** | 0.108, [not significant] | 0.361, [no change] |

^a^-AUT(-Pb), n=11; Control (-Pb), n=6

^b^-AUT(-Pb), n=11; AUT(+Pb), n=4

^MW^- Mann-Whitney test

*, p < 0.05; **, p < 0.01; †, p < 0.1 (trend)

B.

|  | **Proton Pump Inhibitors (PPI)** | |
| --- | --- | --- |
| **Variable** | **AUT(-PPI) vs. Control(-PPI)^a^, p-value^MW^, [effect in AUT(-PPI)]** | **AUT(-PPI) vs. AUT(+PPI)^b^, p-value^MW^, [effect in AUT(+PPI)]** |
| **SI** | 0.003**, [decreased] | 0.794, [no change] |
| **MGAM** | 0.006**, [decreased] | 0.695, [no change] |
| **LCT** | 0.234, [no change] | 0.192, [no change] |
| **SGLT1** | 0.036*, [decreased] | 0.896, [no change] |
| **GLUT2** | 0.036*, [decreased] | 0.602, [no change] |
| **Bacteroidetes IL(RT)** | 0.002**, [decreased] | 0.433, [no change] |
| **Bacteroidetes CEC(RT)** | 0.011*, [decreased] | 0.433, [no change] |
| **Bacteroidetes IL(454)** | 0.036*, [decreased] | 0.050†, [decreased] |
| **Bacteroidetes CEC(454)** | 0.036*, [decreased] | 0.514, [no change] |
| **Firm./Bacteroid. Ratio IL(RT)** | 0.004**, [increased] | 0.602, [no change] |
| **Firm. /Bacteroid. Ratio CEC(RT)** | 0.011*, [increased] | 0.896, [no change] |
| **Firm./Bacteroid. Ratio IL(454)** | 0.027*, [increased] | 0.514, [no change] |
| **Firm./Bacteroid. Ratio CEC(454)** | 0.036*, [increased] | 0.514, [no change] |
| **Clostridiales/Bacteroidales IL(454)** | 0.015*, [increased] | 0.514, [no change] |
| **Clostridiales/Bacteroidales CEC(454)** | 0.036*, [increased] | 0.514, [no change] |
| **Beta-proteobacteria CEC(454)** | 0.047*, [increased] | 0.794, [no change] |

^a^-AUT(-PPI), n=11; Control(-PPI), n=5

^b^-AUT(-PPI), n=11; AUT(+PPI), n=4

^MW^- Mann-Whitney test

*, p < 0.05; **, p < 0.01; †, p < 0.1 (trend)

C.

|  | **Including Antibiotic User (Ab)** | **Excluding Antibiotic User (Ab)** |
| --- | --- | --- |
| **Variable** | **AUT (-Ab) vs. Control (+Ab and -Ab)^a^, p-value^MW^, [effect in AUT(-Ab)]** | **AUT (-Ab) vs. Control (-Ab)^b^, p-value^MW^, [effect in AUT(-Ab)]** |
| **SI** | 0.001**, [decreased] | 0.003**, [decreased] |
| **MGAM** | 0.003**, [decreased] | 0.010**, [decreased] |
| **LCT** | 0.032*, [decreased] | 0.062†, [decreased] |
| **SGLT1** | 0.008**, [decreased] | 0.020*, [decreased] |
| **GLUT2** | 0.010*, [decreased] | 0.024*, [decreased] |
| **Bacteroidetes IL(RT)** | 0.003**, [decreased] | 0.0005***, [decreased] |
| **Bacteroidetes CEC(RT)** | 0.022*, [decreased] | 0.002**, [decreased] |
| **Bacteroidetes IL(454)** | 0.012*, [decreased] | 0.005**, [decreased] |
| **Bacteroidetes CEC(454)** | 0.008**, [decreased] | 0.008**, [decreased] |
| **Firm./Bacteroid. Ratio IL(RT)** | 0.0006***, [increased] | 0.001**, [increased] |
| **Firm. /Bacteroid. Ratio CEC(RT)** | 0.022*, [increased] | 0.008**, [increased] |
| **Firm./Bacteroid. Ratio IL(454)** | 0.026*, [increased] | 0.013*, [increased] |
| **Firm./Bacteroid. Ratio CEC(454)** | 0.032*, [increased] | 0.029*, [increased] |
| **Clostridiales/Bacteroidales IL(454)** | 0.012*, [increased] | 0.008**, [increased] |
| **Clostridiales/Bacteroidales CEC(454)** | 0.032*, [increased] | 0.024*, [increased] |
| **Beta-proteobacteria CEC(454)** | 0.038*, [increased] | 0.120, [no change] |

^a^-AUT(-Ab), n=15; Control(+Ab and -Ab), n=7

^b^-AUT(-Ab), n=15; Control(-Ab), n=6

^MW^- Mann-Whitney test

*, p < 0.05; **, p < 0.01; ***, p < 0.001; †, p < 0.1 (trend)
