# Supplementary material for: Impaired Carbohydrate Digestion and Transport and Mucosal Dysbiosis in the Intestines of Children with Autism and Gastrointestinal Disturbances
Source: PLoS One. 2011 Sep 16;6(9):e24585. doi: 10.1371/journal.pone.0024585 (PMC3174969; doi:10.1371/journal.pone.0024585)
Supplement: Text S1 — Supporting Results. (DOC) [file pone.0024585.s015.doc]

## Text S1: Supporting Results

Genetically determined lactase non-persistence is not responsible for deficient lactase mRNA in AUT-GI children (Figure S1):

Although it is beyond the scope of this study to evaluate all possible mutations in carbohydrate genes that may affect expression, we have confirmed that deficient LCT mRNA in AUT-GI children is not a result of the common adult-type hypolactasia genotype. LCT mRNA levels can be affected by two single nucleotide polymorphisms that determine adult-type hypolactasia; therefore, we genotyped these children using PCR-RFLP analysis. The homozygous, hypolactasia variant alleles were found in 20% (3 out of 15) of AUT-GI children and 14.3% (1 out of 7) of Control-GI children. Genotype proportions were not significantly different between the two groups (chi-squared test, *p*=0.896) (**Figure S1A**). LCT mRNA expression was significantly lower in individuals with the homozygous hypolactasia genotype compared to all other genotypes (**Figure S1B**: Mann-Whitney, *p*= 0.033). Comparison of LCT mRNA expression across genotype and group failed to reach significance (**Figure S1C**: Kruskal-Wallis, *p*=0.097). Comparison of mRNA expression in subjects carrying at least one copy of the normal allele confirmed a significant decrease in LCT mRNA in AUT-GI relative to Control-GI subjects, independent of the individuals with the homozygous hypolactasia genotype (**Figure S1D**: Mann-Whitney, *p*=0.025). In summary, although our data support the notion that LCT genotype affects gene expression, deficient LCT mRNA in AUT-GI was not attributable to disproportionate hypolactasia genotypes between the AUT-GI and Control-GI groups.

Barcoded 16S rRNA gene pyrosequencing (Figure S3):

A total of 525,519 sequencing reads (representing 85% of the initial number of sequencing reads) remained after filtering based on read length, removing low-quality sequences, and combining duplicate pyrosequencing runs (271,043 reads for ilea; 254,476 reads for ceca). Binning of sequences by barcode revealed similar numbers of 16S rRNA gene sequence reads per patient (average # sequences per patient +/- STD for ilea = 12,320 +/-1220; average # sequences per patient +/- STD for ceca = 11,567 +/- 1589). There was not a significant difference between the AUT-GI and Control-GI groups in terms of the number of reads per patient. In order to assess whether sufficient sampling was achieved in the total pyrosequencing data set for all AUT-GI and Control-GI subjects, OTUs (Operational Taxonomic Units) were defined at a threshold of 97% identity, split by data for ileum and cecum, and rarefaction analysis was carried out (**Figure S3A, B**). Rarefaction curves showed a tendency toward reaching plateau for all subjects; however failure to reach plateau suggests that additional sampling would be required to achieve complete coverage of all OTUs present in ileal and cecal biopsies. Investigation of diversity in AUT-GI and Control-GI patients was carried out using the Shannon Diversity Index calculated from OTU data for each subject. Rarefaction analysis revealed that all Shannon Diversity estimates had reached stable values (**Figure** **S3C, D**). While Shannon Diversity estimates varied widely between individuals, there was not an apparent overall difference (loss or gain of diversity) between the AUT-GI and Control-GI groups in ileal (**Figure S3C**) or cecal (**Figure S3D**) biopsies.

**OTU Analysis of Bacteroidetes (Figure S4):**

In order to determine whether the decreased abundance of Bacteroidete members was attributable to the loss of specific Bacteroidete phylotypes, we investigated the distribution of Bacteroidete OTUs (defined using a threshold of 97% identity or greater; 3% distance). The number of Bacteroidete OTUs per patient ranged from 23 to 102 for ileal samples and 10 to 130 for cecal samples. Interestingly, no single OTU was significantly over- or under-represented between AUT-GI and Control-GI children and many OTUs contained single sequences. Furthermore, high inter-subject variability in the distribution and abundance of individual Bacteroidete phylotypes was observed, as has been previously described [1]. Thus, we sought to determine whether the decrease in Bacteroidete abundance in AUT-GI children could be attributed to overall losses of the most prevalent Bacteroidete phylotypes. In both ileal and cecal samples, 12 OTUs accounted for the majority of Bacteroidete sequences (**Figure S4A, B**). The cumulative levels of these 12 OTUs were significantly lower in AUT-GI compared to Control-GI children in both the ileum (**Figure S4C**: Mann-Whitney, *p*=0.008) and cecum (**Figure S4D**: Mann-Whitney, *p*=0.008). Representative sequences from each of these 12 OTUs were classified using Greengenes Blast and microbial blast alignment (NCBI) (**Figure S4E**). The majority of sequences were members of the family Bacteroidaceae (OTUs 1, 3, 5, 6, 7, and 19), except in the case of patient #20, where Prevotellaceae were the dominant phylotype (OTU #21). These results suggest that the loss of Bacteroidetes in AUT-GI children is primarily attributable to overall decreases in the dominant phylotypes of Bacteroidetes in individual patients.

Evaluation of confounding effects of probiotic, proton-pump inhibitor, and antibiotic use:

The use of probiotics (Pb), proton-pump inhibitors (PPI), and antibiotics are reported to exert effects on the composition of the intestinal microbiota [2,3]. As some patients in both the AUT-GI and Control-GI groups had taken these medications, we sought to determine whether potential confounding effects of these medications on our findings could be excluded. Probiotics had been used by both AUT-GI (n=4; 27%) and Control-GI (n=1; 14%) children. If probiotic use determined the outcome of gene expression and bacterial variables, then the significant effect for a given variable should not be present when comparing individuals who had not taken probiotics in the AUT-GI and Control-GI groups [Table S5A: AUT(-Pb) vs. Control(-Pb)]. For each of the 16 variables, except the ratio of Firmicutes to Bacteroidetes in the cecum (RT) and Betaproteobacteria in the cecum (454), either a significant result or trend was observed between the AUT(-Pb) and Control(-Pb) groups. If the cecal ratio of Firmicutes to Bacteroidetes and Betaproteobacteria are affected by probiotic use, then a difference in the levels of these bacterial parameters should be evident when comparing AUT-GI probiotic non-users vs. AUT-GI probiotic users [Table S5A: AUT(-Pb) vs. AUT(+Pb)]. There was not a significant difference in Betaproteobacteria levels between these groups; however, the ratio of Firmicutes to Bacteroidetes in the cecum, determined by real-time PCR, was significantly higher in the AUT(+Pb) group compared to the AUT(-Pb) group (Table S5A: Mann-Whitney, p=0.037). Thus, we cannot exclude a potential effect mediated by probiotics on this variable. This effect, however, was not apparent in the corresponding ratio of Firmicutes to Bacteroidetes in the cecum, as determined by pyrosequencing.

The use of proton-pump inhibitors (PPI: Lansoprazole or Omeprazole) was similarly examined. PPI had been used by both AUT-GI (n=4; 27%) and Control-GI (n=2; 29%) children. A significant difference was found for all variables, except LCT, when comparing AUT(-PPI) children with Control(-PPI) children [Table S5B: AUT(-PPI) vs. Control(-PPI)]. Thus, a potential effect of PPI use should only be considered for LCT. As LCT levels were not significantly different between AUT(-PPI) and AUT(+PPI) children, it is unlikely that PPIs exerted any major effect on LCT expression. A trend toward an effect in the levels of Bacteroidetes in the ileum, as determined by pyrosequencing, was evident between AUT(-PPI) and AUT(+PPI) children; however, a significant effect was observed between AUT(-PPI) and Control(-PPI) children. This suggests that this potential effect was not a major determinant of the difference in ileal Bacteroidetes between AUT-GI and Control-GI children. Only one patient (AUT-GI patient #1) had used both probiotics and proton-pump inhibitors, thus an additive effect was not evaluated. Grouping of patients based on whether they had taken either probiotics or proton-pump inhibitors did not reveal any significant effects in the 16 variables (data not shown).

Only one individual had taken an antibiotic (amoxicillin) in this cohort (Control-GI patient # 16). This patient had high levels of mRNA expression for all disaccharidases and transporters, within the range of other Control-GI children and at least above the 90^th^ percentile of all AUT-GI children. Thus, exclusion of this patient from the analysis had a negative effect on significance values obtained for gene expression assays (**Table S5C**). These results suggest that antibiotic use had no effect on disaccharidase and hexose transporter levels in this patient. In contrast, Control-GI patient #16 consistently had the lowest levels of Bacteroidetes (representing the low-range outlier) compared to all other Control-GI children in pyrosequencing and real-time PCR assays. Thus, exclusion of this patient from analysis of bacterial phylotypes generally increased the significance of results obtained for Bacteroidetes, ratios of Firmicutes to Bacteroidetes, and ratios of Clostridiales to Bacteroidales. Levels of Betaproteobacteria in the cecum for this patient were near the median value of all other Control-GI children. Thus, it is likely that antibiotic use in this patient had some effect on Bacteroidete levels, but no effect on Betaproteobacteria or gene expression for disaccharidases and transporters. As antibiotic use in this patient did not affect all variables and exclusion of this patient did not affect the interpretation of results, we have not excluded this patient from our overall analysis.

**Text S1 References**

1. Eckburg PB, Bik EM, Bernstein CN, Purdom E, Dethlefsen L, et al. (2005) Diversity of the human intestinal microbial flora. Science 308: 1635-1638.

2. Reid G, Younes JA, Van der Mei HC, Gloor GB, Knight R, et al. (2011) Microbiota restoration: natural and supplemented recovery of human microbial communities. Nat Rev Microbiol 9: 27-38.

3. Lombardo L, Foti M, Ruggia O, Chiecchio A (2010) Increased incidence of small intestinal bacterial overgrowth during proton pump inhibitor therapy. Clin Gastroenterol Hepatol 8: 504-508.
