# Supplementary material for: Impaired Carbohydrate Digestion and Transport and Mucosal Dysbiosis in the Intestines of Children with Autism and Gastrointestinal Disturbances
Source: PLoS One. 2011 Sep 16;6(9):e24585. doi: 10.1371/journal.pone.0024585 (PMC3174969; doi:10.1371/journal.pone.0024585)
Supplement: Text S2 — Supporting Methods. (DOC) [file pone.0024585.s016.doc]

**Text S2: Supporting Methods**

**Quantitative Real-Time PCR of human mRNA:**

PCR standards for determining copy numbers of target transcripts were generated from amplicons of SI, MGAM, LCT, SGLT1, GLUT2, Villin, CDX2, GAPDH, and Beta-actin cloned into the vector pGEM-T easy (Promega Corporation). Linearized plasmids were quantitated using a Nanodrop ND-1000 Spectrophotometer, and 10-fold serial dilutions (ranging from 5 x 10^5^ to 5 x 10^0^ copies) were created in water containing yeast tRNA (1 ng/µl). Unpooled RNA from individual ileal biopsies was used for real-time PCR assays; each individual biopsy was assayed in duplicate. cDNA was synthesized using Taqman reverse transcription reagents (Applied Biosystems) from 2 µg unpooled RNA per 100 µl reaction. Each 25-µl amplification reaction contained 10 µl template cDNA, 12.5 µl Taqman Universal PCR Master Mix (Applied Biosystems), 300 nM gene-specific primers and 200 nM gene-specific probe (**Table S6**). The thermal cycling profile using a ABI StepOnePlus Real-time PCR System (Applied Biosystems) consisted of: Stage 1, one cycle at 50o C for 2 min; Stage 2, 1 cycle at 95o C for 10 min; Stage 3, 45 cycles at 95o C for 15 s and 60o C for 1 min (1 min 30 s for LCT). GAPDH and B-actin mRNA were amplified in duplicate reactions by real-time PCR from the same reverse transcription reactions as were used for the genes of interest. The mean concentration of GAPDH or Beta-actin in each sample was used to control for integrity of input RNA and to normalize values of target gene expression to those of the housekeeping gene expression. GAPDH and Beta-actin have been shown to be the most stable reference genes in small bowel and colonic biopsies from healthy patients and pediatric patients with inflammatory bowel disease [1]. The final results shown were expressed as the mean copy number from replicate biopsies per patient, relative to values obtained for GAPDH mRNA. Beta-actin normalization gave similar results to GAPDH normalization for all assays (data not shown). Due to insufficient or poor quality RNA (OD 260/280 ratio < 1.7, or RNA integrity number < 7.0), only 3 of the 4 biopsies were included for 3 patients (patients #4, 7, 10) and only 2 of the 4 biopsies were included for 1 patient (patient #2). Thus, 83 of the original 88 ileal biopsies were used in real-time PCR experiments.

**Lactase genotyping:**

Genotyping primers for the LCT C/T-13910 and G/A-22018 polymorphisms are as follows: C/T-13910For (5’-GGATGCACTGCTGTGATGAG-3’), C/T-13910Rev (5’-CCCACTGACCTATCCTCGTG-3’), G/A-22018For (5’-AACAGGCACGTGGAGGAGTT-3’), and G/A-22018Rev (5’-CCCACCTCAGCCTCTTGAGT-3’). Each 50-μl amplification reaction contained 500 ng genomic DNA, 400 nM forward and reverse primers, and 25 μl High Fidelity PCR master mix (Roche). Thermal cycling consisted of 1 cycle at 94^o^C for 4 min followed by 40 cycles at 94^o^C for 1 min, 60^o^C for 1 min, and 72^o^C for 1 min. PCR reactions for C/T-13910 were directly digested with the restriction enzyme BsmFI at 65^o^C for 5 hrs. PCR reactions for G/A-22018 were resolved on 1% agarose gels followed by gel extraction of the prominent 448bp amplicon. Gel extracted G/A-22018 amplicons were then digested with the restriction enzyme HhaI at 37^o^C for 5 hrs. Restriction digests of C/T-13910 and G/A-22018 were resolved on 1.5% ethidium-stained agarose gels for genotyping analysis. BsmFI digestion of the C/T-13910 amplicons generates two fragments (351bp and 97bp) for the hypolactasia genotype (C/C), four fragments (351bp, 253bp, 98bp, and 97bp) for the heterozygous genotype (C/T), and three fragments (253bp, 98bp, and 97bp) for the normal homozygous allele (T/T). HhaI digestion of the G/A-22018 amplicons generates two fragments (284bp and 184bp) for the hypolactasia genotype (G/G), three fragments (448bp, 284bp, and 184bp) for the heterozygous genotype (G/A), and a single fragment (448bp) for the normal homozygous allele (A/A).

**Barcoded pyrosequencing of intestinal microbiota:**

Composite primers used for pyrosequencing analysis were as follows: (For) 5’-GCCTTGCCAGCCCGCTCAGTCAGAGTTTGATCCTGGCTCAG-3’, (Rev) 5’-GCCTCCCTCGCGCCATCAGNNNNNNNNCATGCTGCCTCCCGTAGGAGT-3’. Underlined sequences in the Forward and Reverse primers represent the 454 Life Sciences@ primer B and primer A, respectively. Bold sequences in the forward and reverse primers represent the broadly-conserved bacterial primer 27F and 338R, respectively. NNNNNNNN represents the eight-base barcode, which was unique for each patient. PCR reactions consisted of 8 μl 2.5X 5 PRIME HotMaster Mix (5 PRIME Inc), 6 μl of 4 μM forward and reverse primer mix, and 200 ng DNA in a 20-μl reaction volume. Thermal cycling consisted of one cycle at 95^o^ C for 2 min; and 30 cycles at 95^o^ C for 20 seconds, 52^o^ C for 20 seconds, and 65^o^ C for 1 min. Each of 4 biopsies per patient was amplified in triplicate, with a single, distinct barcode applied per patient. Ileal and cecal biopsies were assayed separately. Reagent controls were included (negative controls) to control for any background contamination. Triplicate reactions of individual biopsies and reagent controls were combined, and PCR products were purified using Ampure magnetic purification beads (Beckman Coulter Genomics) and quantified with the Quanti-iT PicoGreen dsDNA Assay Kit (Invitrogen) and Nanodrop ND-1000 Spectrophotometer (Nanodrop Technologies). Equimolar ratios were combined to create two master DNA pools, one for ileum and one for cecum, with a final concentration of 25 ng/μl. Master pools were sent for unidirectional pyrosequencing with primer A at 454 Life Sciences on a GS FLX sequencer. Each master pool was sequenced in duplicate on different days to control for variability in the sequencing reactions. Sequences obtained from duplicate runs were combined for the final analysis. No sequences were obtained from reagent controls, indicating that no background contamination was present.

Quantitative Real-time PCR of Bacteroidete and Firmicute 16S rRNA genes:

PCR standards for determining copy numbers of bacterial 16S rDNA were prepared from representative amplicons of the partial 16S rRNA genes of Bacteroidetes, Firmicutes, and total Bacteria cloned into the vector PGEM-T easy (Promega). A representative amplicon with high sequence similarity to *Bacteroides Vulgatus* (Accession #: NC_009614) was used with Bacteroidete-specific primers. A representative amplicon with high sequence similarity to *Faecalibacterium prausnitzii* (Accession #: NZ_ ABED02000023) was used with Firmicute-specific primers. A representative amplicon with high sequence similarity to *Bacteroides intestinalis* (Accession #: NZ_ABJL02000007) 16S rRNA gene was used with total Bacteria primers (primers 515F and 805R). Cloned sequences were classified using the Ribosomal Database Project (RDP, release 10) Seqmatch tool and confirmed by the Microbes BLAST database. Plasmids were linearized with the SphI restriction enzyme, quantitated, and ten-fold serial dilutions of plasmid standards were created ranging from 5X10^7^ to 5x10^0^ copies for Bacteroidetes, Firmicutes, and total Bacteria. Amplification and detection of DNA by real-time PCR were performed with the ABI StepOnePlus Real-time PCR System (Applied Biosystems). Cycling parameters for Bacteroidetes and total Bacteria were as previously described [2], as were cycling parameters for Firmicutes [3]. Each 25-μl amplification reaction mixture contained 50 ng DNA, 12.5 μl SYBR Green Master Mix (Applied Biosystems), and 300 nM bacteria-specific (Bacteroidete, Firmicute, or total Bacteria) primers. DNA from each of 88 ileal biopsies (4 biopsies per patient) and 88 cecal biopsies (4 biopsies per patient) was assayed in duplicate. The final results were expressed as the mean number of Bacteroidete or Firmicute 16S rRNA gene copies normalized to 16S rRNA gene copies obtained using total Bacterial primers. Eight water/reagent controls were included for all amplifications. The average copy number for water/reagent controls (background) was subtracted from each ileal and cecal amplification prior to normalization. For the Bacteroidete assay all water/reagent controls contained undetectable levels of amplification. For the Firmicute assay average amplification signal from water/reagent controls were minimal, 12.03 +/- 15.0 copies.

**Statistical analysis:**

To evaluate the effects of CDX2 and/or villin on enzyme and transporter levels and the effects of levels of enzymes and transporters on bacterial levels, multiple linear regression analyses were conducted. For assessing the effects of CDX2 and villin on disaccharidase and transporter expression levels, disaccharidase and transporter levels were log-transformed to stabilize the variance. Using each log-transformed disaccharidase and transporter mRNA expression level as an outcome, three models were fitted: first, with CDX2 only as independent variable; second, with CDX2 and diagnostic status (dummy coded; AUT-GI = 1 vs. Control-GI = 0); and third, with CDX2, diagnostic status, and the interaction term between CDX2 and diagnostic status. The interaction term allowed us to evaluate whether the effect of CDX2 on disaccharidases and transporters was similar for AUT-GI and Control-GI children. The same models were fitted after adding villin and the interaction term between villin and diagnostic status. The coefficient estimates in **Table 3** represent change in log-transformed disaccharidase or transporter mRNA levels per unit standard deviation increase in CDX2 and villin mRNA levels.

To delineate the effects of disaccharidases and transporters on bacterial levels in ileal and cecal biopsies, bacterial 16S rRNA gene quantities (obtained from real-time PCR for Bacteroidetes and Firmicutes) or abundance (obtained from 454 pyrosequencing data for Proteobacteria and Betaproteobacteria) were log-transformed to stabilize variance. For each of the log-transformed bacterial levels, we first fitted enzyme levels simultaneously as the main effects (SI, MGAM, LCT, SGLT1, and GLUT2) to evaluate the effects of enzymes on a given bacterial taxa. Diagnostic status was added to the model to determine whether there was a residual difference in bacterial levels between AUT-GI and Control-GI children after adjusting for the levels of disaccharidases and transporters. We further examined whether the effect of disaccharidases or transporters on bacterial levels was the same depending on the diagnostic status by examining two-way interaction terms between diagnostic status and each disaccharidase and transporter. The final model was derived by including all the main effect terms and selectively including two-way interaction terms using the backward elimination method starting from all possible two-way interaction terms with diagnostic status and the individual disaccharidases and transporters. The coefficient estimates in **Table 4** represent change in log-transformed bacterial levels per unit standard deviation increase in disaccharidase or transporter levels. The statistical package R (version 2.7.0) was used for regression analysis.

**Text S2 References**

1. Zilbauer M, Jenke A, Wenzel G, Goedde D, Postberg J, et al. (2010) Intestinal alpha-defensin expression in pediatric inflammatory bowel disease. Inflamm Bowel Dis. In press.

2. Frank DN, St Amand AL, Feldman RA, Boedeker EC, Harpaz N, et al. (2007) Molecular-phylogenetic characterization of microbial community imbalances in human inflammatory bowel diseases. Proc Natl Acad Sci U S A 104: 13780-13785.

3. Guo X, Xia X, Tang R, Zhou J, Zhao H, et al. (2008) Development of a real-time PCR method for Firmicutes and Bacteroidetes in faeces and its application to quantify intestinal population of obese and lean pigs. Lett Appl Microbiol 47: 367-373.
